# What we can learn from nature to restore human vision

**DOI:** 10.3389/fnhum.2026.1968351

**Published:** 2026-10-20

**Authors:** Marianna Weener, Lusine Khachatryan, Boris Baziyan

**Affiliations:** 1 Broad Institute, Cambridge, MA, United States; 2 Pediatric Neurology Department, N.F. Filatov Clinical Institute of Children's Health, Sechenov University, Moscow, Russia; 3 Neurocybernetics Lab, Russian Center of Neurology and Neurosciences, Moscow, Russia

**Keywords:** bio-inspired devices, comparative visual neuroscience, gene therapy, optogenetics, retinal prosthesis, retinal regeneration, vision restoration

## Abstract

Irreversible vision loss, affecting millions of people worldwide, most often ends in the death of retinal neurons that the human eye cannot replace. Across the tree of life, however, evolution has repeatedly produced strategies that address the core problems of vision restoration: zebrafish and salamanders regenerate functional retinas and lenses, and a wide range of species have tuned photoreceptors, optics, and neural circuits to extract maximal information from light. This review systematizes these biological strategies and evaluates how each is being translated toward human therapies, with grading of the strength of evidence behind every translational claim. We organize the field by biological strategy: regeneration, spectral tuning, low-light adaptation, high-acuity optics, polarization and additional channels, and neural processing, and for each trace the path from mechanism to therapy or device. The most mature translations are already clinical: gene therapy for inherited retinal disease, and optogenetic restoration of vision in blind patients. Regenerative approaches informed by regenerating species, including Müller glia reprogramming, ciliary marginal zone biology, retinal organoids, and stem cell transplantation, are advancing toward clinical feasibility. Spectral, low-light, and acuity specializations provide mechanistic blueprints and bio-inspired devices, though their biological translation to humans remains preclinical or theoretical. We conclude with the controversies, research gaps, and ethical considerations that will shape whether nature's strategies can be safely and equitably applied to restore human vision, and we briefly consider the evidence and limits of extending vision beyond its native range.

## 1 Introduction

Vision is the dominant human sense, yet it is also one of the most fragile. An estimated 2.2 billion people worldwide live with some form of vision impairment, and for the leading causes of irreversible blindness: age-related macular degeneration, retinitis pigmentosa, glaucoma, and diabetic retinopathy, the common final pathway is the death of retinal neurons or photoreceptors that the human eye cannot replace (Burton et al., 2021; Bourne et al., 2020). The central question motivating this review is how the biological strategies that evolution has produced across the animal kingdom can be harnessed to restore vision that has been lost.

To frame that question, we first briefly summarize the structure and development of the retina, because the translational barriers discussed throughout this review are rooted in retinal anatomy and developmental history. The vertebrate retina is a layered neural tissue built from a conserved set of cell types: rod and cone photoreceptors (PRs) that capture light; interneurons—bipolar cells (BPCs), horizontal cells (HCs), and amacrine cells (ACs), that process the signal; retinal ganglion cells (RGCs) whose axons form the optic nerve and carry the output to the brain; Müller glia (MG), the principal radial glial cell type that spans the retinal layers and supports neuronal homeostasis; and the retinal pigment epithelium (RPE) that sustains the photoreceptors (Livesey and Cepko, 2001; Bassett and Wallace, 2012). All of these cell types arise during development from a common pool of multipotent retinal progenitor cells under the control of a conserved gene-regulatory network (including *Pax6, Six3, Six6*, and the proneural factors; Cepko, 2015). Müller glia is among the last cell types to be born, share a lineage with retinal neurons, and retain a molecular profile closer to retinal progenitors than any other mature retinal cell. This is a developmental heritage that underlies their latent regenerative competence in non-mammalian vertebrates (Goldman, 2014). In mammals, retinal injury triggers MG to undergo reactive gliosis: hypertrophy, upregulation of intermediate filaments, and limited, non-productive cell-cycle re-entry rather than the dedifferentiation and neurogenesis seen in fish and amphibians (Lahne et al., 2020; Goldman, 2014). This developmental and injury-response context is essential for understanding why some species regenerate and others do not, and where the molecular levers for intervention lie (Table 1).

**Table 1:** Model species, visual specializations, key mechanisms, and translational relevance.

Species/group	Visual specialization	Key mechanism	Translational relevance	Evidence	References
Zebrafish (^*^Danio rerio^*^)	Full retinal regeneration	Müller glia dedifferentiation via *Ascl1a/Lin-28; TGF-β*, Yap, inflammation; persistent CMZ stem cells	Endogenous retinal repair; Müller glia reprogramming; CMZ biology	Preclinical	Powell et al., 2016; Celotto et al., 2023; Lyu et al., 2023; Ramachandran et al., 2010; Fausett and Goldman, 2006; Sharma et al., 2020; Lourenço et al., 2021; Nagashima and Hitchcock, 2021; Lee et al., 2023; Fischer et al., 2013
Salamander/newt/axolotl	Lens & neural retina regeneration	Iris/RPE pigment epithelial cell transdifferentiation via a multipotent intermediate; *Tbx5, Fgf10, Pax6*, choroid signals	Cell-fate conversion; RPE/lens/retina regeneration	Preclinical	Tsonis et al., 2004; Sousounis et al., 2013; Henry and Hamilton, 2018; Suetsugu-Maki et al., 2012; Tsissios et al., 2023; Islam et al., 2014; Grigoryan and Markitantova, 2016; Mitsuda et al., 2005
^*^Xenopus^*^ frog	Whole-retina regeneration	Ciliary marginal zone (CMZ) stem/progenitor cells; RPE transdifferentiation post-metamorphosis	Retinal stem cell niche; CMZ reactivation	Preclinical	Miyake and Araki, 2014; Xue and Harris, 2012; Ail and Perron, 2017; Frøen et al., 2013; Islam et al., 2014
Chick (embryonic)	Partial retinal regeneration	Müller glia → MGPCs via FGF/MAPK; BMP (+), TGF-β (–), NFI (blocks); residual CMZ	Signaling levers for glia-to-neuron conversion	Preclinical	Fischer et al., 2008; Todd et al., 2017; El-Hodiri et al., 2021; Campbell et al., 2021; Kelly et al., 2024
Planarian flatworm	Whole-eye regeneration	Pluripotent neoblasts → lineage-restricted optic progenitors; *ovo/six1/2/eya, otxA, sp6-9/dlx; Pax6*-independent	*De novo* eye organogenesis from stem cells	Theoretical	Wagner et al., 2011; Reddien, 2012; Lapan and Reddien, 2011; LoCascio et al., 2017; Scimone et al., 2026, 2025
Mouse (mammal)	No spontaneous regeneration	Müller glia reactive gliosis; reprogrammable via Ascl1 + HDACi/AAV proneural factors; quiescent ciliary-body progenitors reactivatable via *BMP/sFRP2* antagonism	Mammalian Müller glia reprogramming; endogenous progenitor reactivation	Preclinical	Jorstad et al., 2017; Todd et al., 2021, 2022; Pavlou et al., 2025; Jorstad et al., 2020; Xiao et al., 2023; Tropepe et al., 2000; Zhao et al., 2005; Abdouh and Bernier, 2006; Grise et al., 2021
Owl	Scotopic low-light vision	Rod-dominated retina (~30:1 rods:cones), high sensitivity, scotopic acuity	Low-light sensor design; scotopic restoration	Preclinical	Braekevelt, 1993; Orlowski et al., 2012; Wu et al., 2016; Borges et al., 2019
Deep-sea fish (spinyfin, lantern fish)	Extreme low-light & multi-opsin rods	Multiple rod opsins, rod convergence, tapetum, tubular eyes	Photon-capture limits; scotopic spectral tuning	Preclinical	de Busserolles et al., 2020; Musilová et al., 2018; de Busserolles et al., 2014; de Busserolles and Justin Marshall, 2017; Fogg et al., 2026; Hofmann and Lamb, 2022
Eagle/falcon (raptor)	Highest acuity (~2 × human), double fovea	High foveal cone density, tubular eye, large pupil, tetrachromacy	High-acuity optics; multifocal IOL/implant design	Clinical (optics)	Mitkus et al., 2017a; González-Martín-Moro et al., 2017; Mitkus et al., 2017b; Zueva et al., 2014; Potier, 2020; Potier et al., 2017
Four-eyed fish (^*^Anableps^*^)	Simultaneous aerial + aquatic vision	Dual optical axes in one eye; ≥10 opsins; ventral retina specialization	Dual-mode optics; multifocal design	Theoretical	Schwab, 2002; Perez et al., 2017; Owens et al., 2009
Bird (passerine, etc.)	UV vision, tetrachromacy	SWS1 UV opsin + UV-transmitting ocular media; SWS2 carotenoid tuning	UV optics/opsin engineering; spectral restoration	Clinical (UV-IOL)	Cronin and Bok, 2016; Peng et al., 2024; Toomey et al., 2016; Hogg et al., 2011; Ödeen et al., 2011; Ödeen and Håstad, 2013
Reindeer	UV vision in Arctic winter	UV-permissive cornea/lens (not a novel opsin); UV contrast for forage in snow	Optical spectral extension; ocular-media engineering	Theoretical	Olsson et al., 2021; Lind et al., 2013; Tyler et al., 2014; Dominy et al., 2023
Pit viper/boa/python	Infrared detection	Warmth-activated TRPA1 in pit organ; convergent molecular evolution	IR sensing; thermal imagers; upconversion	Preclinical	Gracheva et al., 2010; Panzano et al., 2010; Yokoyama et al., 2010; Geng et al., 2011
Mantis shrimp	12–16 spectral channels + polarization	Many photoreceptor classes + filtering pigments; scanning color recognition; polarization receptors	Multispectral & polarization sensors; efficient coding	Preclinical	Cronin et al., 2014; Thoen et al., 2014; Wang and Marshall, 2024; Liu et al., 2022
Cat	Nocturnal sensitivity	Tapetum lucidum, high rod density, summation	Low-light sensor design; photon recycling	Preclinical	Vee et al., 2022; Schwab et al., 2002
Algae (e.g., ^*^Chlamydomonas^*^)	Light-gated ion channels	Channelrhodopsin directly light-activated cation channel	Optogenetic vision restoration	Clinical	Sahel et al., 2021; Mohanty et al., 2025; Fong et al., 2025; Poboży et al., 2025; Bansal et al., 2021; Alekseev et al., 2025; Ganjawala et al., 2019; Lu et al., 2024; Sakai et al., 2022
Human (reference)	Trichromatic, ~380–740 nm, no regeneration	Three cone opsins; UV-blocking lens; limited scotopic sensitivity	Baseline for restoration	—	Livesey and Cepko, 2001; Cronin and Bok, 2016; Jordan et al., 2010

Nature has already run the relevant experiments. Over roughly 500 million years, eyes have evolved independently dozens of times, and extant species collectively demonstrate strategies that address nearly every limitation of the human visual system (Nilsson, 2013). Some vertebrates can regenerate the retina or lens after catastrophic injury, as seen in zebrafish and newts. Others have evolved radically different visual solutions: birds can extend spectral sensitivity into the ultraviolet, pit vipers detect infrared radiation, many birds use four or five spectral channels, and nocturnal or predatory species have optimized their retinas and optics for either extreme sensitivity or exceptional spatial resolution. Others evolved specialized visual systems that detect polarization, combine multiple optical systems, or extract motion and contrast with remarkable efficiency. Mantis shrimp, for example, possess sophisticated polarization-sensitive vision; jumping spiders combine high-resolution principal eyes with motion-sensitive secondary eyes; and dragonflies have neural circuits specialized for the rapid detection and tracking of moving objects. These are working prototypes, refined by selection, that reveal which biological parameters are plastic and which are constrained, and therefore which levers are available to repair a damaged human retina. We note at the outset that evolution does not produce “solutions” in the engineering sense; it selects and preserves strategies that improve survival and reproductive success under specific environmental conditions. We therefore use the term “biological strategy” or “adaptation” throughout (Table 2).

**Table 2:** Translational status of nature-inspired vision restoration approaches.

Approach	Biological inspiration	Target condition/goal	Status	Evidence	Key evidence/outcome	References
Gene augmentation therapy	Native gene function (e.g., RPE65)	RPE65-mediated Leber amaurosis	Approved (FDA/EMA)	Clinical	Phase 3: mean bilateral MLMT gain 1.8 vs. 0.2 light levels; 65% passed lowest luminance	Russell et al., 2017; Graf and Machta, 2024; Sarbout et al., 2025; Lorenz, 2025
Gene therapy achromatopsia	Native cone channel function (CNGA3/CNGB3)	Achromatopsia (cone channelopathy)	Clinical trials	Clinical	Safe; improved acuity/contrast (CNGA3); cone-pathway plasticity in children	Fischer et al., 2020; Reichel et al., 2021; Michaelides et al., 2023; Farahbakhsh et al., 2022
Optogenetic therapy (ganglion cells)	Algal channelrhodopsin	Retinitis pigmentosa (late-stage)	Clinical trials	Clinical	Partial functional recovery: object perception/localization with light-gated goggles (ChrimsonR)	Sahel et al., 2021; Mohanty et al., 2025; Fong et al., 2025; Poboży et al., 2025
Optogenetic therapy (bipolar cells)	Synthetic multi-characteristic opsin (MCO1)	Severe retinal degeneration	Clinical trials	Clinical	Improved shape discrimination & mobility (small open-label)	Mohanty et al., 2025
Subretinal photovoltaic prosthesis (PRIMA)	Photoreceptor-like photovoltaic conversion	Geographic atrophy (dry AMD)	Clinical trials	Clinical	Restored central vision; up to 8 ETDRS lines with zoom at 4 years	Palanker et al., 2020; Muqit et al., 2024; Bhuckory et al., 2025
Epiretinal prosthesis (Argus II)	Electrical stimulation of ganglion cells	End-stage retinitis pigmentosa	Approved (discontinued)	Clinical	Light perception, functional gains in >70% at 2 years (French post-approval)	Delyfer et al., 2020; Bloch and da Cruz, 2019; Finn et al., 2018
Nanowire retinal nanoprosthesis	Photovoltaic nanomaterials	Blindness (preclinical models)	Preclinical	Preclinical	Improved vision in blind mice & non-human primates; visible + NIR response	Wang et al., 2025b
Müller glia reprogramming	Zebrafish/chick regeneration	Retinal degeneration	Preclinical	Preclinical	New retinal neurons in adult mice via Ascl1 + HDACi/AAV proneural factors	Jorstad et al., 2017; Todd et al., 2021, 2022; Pavlou et al., 2025; Jorstad et al., 2020; Xiao et al., 2023
Endogenous progenitor reactivation	Mammalian ciliary-body stem cells	Retinal degeneration	Preclinical	Preclinical	BMP/sFRP2 antagonism reactivates quiescent ciliary epithelium stem cells *in vivo*	Tropepe et al., 2000; Zhao et al., 2005; Abdouh and Bernier, 2006; Grise et al., 2021
Retinal organoid/photoreceptor transplantation	hPSC retinal organoids; regenerative species	Retinal degeneration, retinitis pigmentosa	First-in-human/Preclinical	Clinical (early)/Preclinical	iPSC retinal organoid sheets safe at 2 years; cone transplants restore function in models	Mandai, 2023; Gagliardi et al., 2019; Ribeiro et al., 2021; Léveillard and Klipfel, 2019; Iwama et al., 2025; Zhang and Jin, 2024; da Cruz et al., 2018; Sharma et al., 2019
RPE cell transplantation	Salamander RPE plasticity; stem cell biology	Age-related macular degeneration	Clinical trials (early)	Clinical (early-phase)	hESC/iPSC-RPE patches feasible & safe; acuity gains in some patients	da Cruz et al., 2018; Sharma et al., 2019
RGC transplantation/optic nerve regeneration	Stem cell-derived RGCs; PTEN/SOCS3 biology	Glaucoma, optic neuropathy	Preclinical	Preclinical	Donor RGC integration (ILM disruption); long-distance axon regeneration & target reinnervation	Soucy et al., 2023; Zhang et al., 2021a; Oswald et al., 2021; Agarwal et al., 2023; Aguzzi et al., 2026; Sun et al., 2011; Smith et al., 2009; de Lima et al., 2012; Zhang et al., 2025; Liu et al., 2025
Injectable upconversion nanoantennae	IR detection (snake-inspired concept)	Near-infrared perception	Preclinical (mice)	Preclinical	Lanthanide UCNPs convert NIR to visible; mice perceive NIR patterns	Ma et al., 2019
Upconversion contact lenses	IR detection (bio-inspired photonics)	Near-infrared perception (humans)	Human demonstration	Proof-of-concept	Humans perceive NIR spatiotemporal patterns & NIR “color”	Cai et al., 2019; Do, 2025; Wang et al., 2025a
Biomimetic polarization sensors	Mantis shrimp/insect compound eye	Navigation, machine vision	Devices (prototype)	Preclinical	Full-Stokes, wide-field, ultra-thin polarization imaging	Liu et al., 2022; Dupeyroux et al., 2019; Liu et al., 2023; Miyata et al., 2020
Biomimetic IR imagers	Snake pit organ	Thermal imaging	Devices (prototype)	Preclinical	Uncooled, self-powered mid/long-IR imaging	Zhang et al., 2021b; Kogos et al., 2020
Compound-eye cameras	Insect ommatidia	Wide-field multispectral imaging	Devices (prototype)	Preclinical	Real-time multispectral imaging over ultra-large FOV	Ding et al., 2022; Mu et al., 2025

The translational landscape has changed dramatically in the past decade, and it is now possible to grade the maturity of each nature-inspired approach. Gene therapy for inherited retinal disease is a clinical reality (Russell et al., 2017). Optogenetic restoration of vision is directly inspired by light-gated ion channels from algae and has produced partial functional recovery in blind patients (Sahel et al., 2021). Retinal prostheses have arguably restored reading vision in geographic atrophy (Palanker et al., 2020). Reprogramming of mammalian Müller glia, informed by zebrafish regeneration, has generated new retinal neurons in adult mice (Jorstad et al., 2017; Lahne et al., 2020). And wearable upconversion devices have extended human perception into the near infrared (Cai et al., 2019; Powell et al., 2016). The field has moved from descriptive comparative biology to mechanism-driven engineering, but the maturity of these translations varies enormously from approved therapies to single-patient studies to theoretical proposals.

This review provides a systematic, mechanism-first synthesis of how biological visual specializations can inform the restoration of human vision. We organize the literature by biological strategy, because the same mechanism (for example, opsin spectral tuning) can serve multiple translational goals, and because a mechanism-first organization makes the translation logical. For each strategy we describe the biological adaptation and its mechanistic basis, evaluate the current state of translation to humans, and identify the limiting constraints. To counter the risk of overstatement that is inherent in a field spanning approved therapies and speculative proposals, we assign every translational claim an evidence grade. We define four grades: **Clinical** (tested in human patients with a measured functional outcome), **Preclinical**—demonstrated in a whole-animal model with a functional or histological readout, **Proof-of-concept**—demonstrated in principle, typically in a single study, a small case series, or an *in vitro*/*ex vivo* preparation, and **Theoretical**—proposed on mechanistic grounds but not yet demonstrated in a living system (Table 3).

**Table 3:** Evidence strength of nature-inspired vision restoration strategies.

Biological strategy	Biological source	Translational approach	Evidence grade	References
Retinal regeneration (Müller glia)	Zebrafish, chick	Müller glia reprogramming (Ascl1, AAV proneural factors)	Preclinical	Jorstad et al., 2017; Todd et al., 2021, 2022; Pavlou et al., 2025; Jorstad et al., 2020; Xiao et al., 2023
Retinal regeneration (CMZ)	Fish, amphibian	CMZ stem cell biology; CMZ reactivation	Preclinical/theoretical (mammals)	Fischer et al., 2013; Miles and Tropepe, 2021; Wan et al., 2016; Otteson and Hitchcock, 2003; Miyake and Araki, 2014; Xue and Harris, 2012; Ail and Perron, 2017; Frøen et al., 2013
Endogenous progenitor reactivation	Mammalian ciliary body	BMP/sFRP2 antagonism; growth-factor activation	Preclinical (rodent)/Theoretical (human)	Tropepe et al., 2000; Zhao et al., 2005; Abdouh and Bernier, 2006; Grise et al., 2021
Pigment epithelium transdifferentiation	Salamander, Xenopus, chick	RPE/iris cell-fate conversion	Preclinical	Tsonis et al., 2004; Sousounis et al., 2013; Henry and Hamilton, 2018; Suetsugu-Maki et al., 2012; Tsissios et al., 2023; Islam et al., 2014; Grigoryan and Markitantova, 2016; Mitsuda et al., 2005
Retinal organoids & cell transplantation	hPSC organoids; regenerative species	Photoreceptor/RPE/organoid-sheet transplantation	Clinical (early-phase)/Preclinical	Mandai, 2023; Gagliardi et al., 2019; Ribeiro et al., 2021; Léveillard and Klipfel, 2019; Iwama et al., 2025; Zhang and Jin, 2024; da Cruz et al., 2018; Sharma et al., 2019
RGC replacement & optic nerve regeneration	Stem cell RGCs; PTEN/SOCS3	RGC transplant; axon regeneration & target reinnervation	Preclinical	Soucy et al., 2023; Zhang et al., 2021a; Oswald et al., 2021; Agarwal et al., 2023; Aguzzi et al., 2026; Sun et al., 2011; Smith et al., 2009; de Lima et al., 2012; Zhang et al., 2025; Liu et al., 2025
Spectral restoration (UV)	Birds, reindeer	UV-transmitting intraocular lenses	Clinical	Cronin and Bok, 2016; Orlowski et al., 2012
Spectral restoration (achromatopsia)	Native cone channel	CNGA3/CNGB3 gene therapy	Clinical	Fischer et al., 2020; Reichel et al., 2021; Michaelides et al., 2023; Farahbakhsh et al., 2022
Spectral extension (IR)	Pit viper, bio-inspired photonics	Upconversion nanoparticles/contact lenses	Preclinical (injectable); Proof-of-concept (wearable)	Ma et al., 2019; Do, 2025; Wang et al., 2025a
Tetrachromacy/added channels	Birds, mantis shrimp	Opsin engineering; neural rewiring	Theoretical	Cronin et al., 2014; Jordan et al., 2010
Low-light (scotopic) restoration	Owl, deep-sea fish, cat	Rhodopsin tuning; sensor design; prosthetic coding	Preclinical	Braekevelt, 1993; Orlowski et al., 2012; Wu et al., 2016; Borges et al., 2019; Vee et al., 2022; Schwab et al., 2002; de Busserolles et al., 2020; Musilová et al., 2018; de Busserolles et al., 2014; de Busserolles and Justin Marshall, 2017; Fogg et al., 2026; Hofmann and Lamb, 2022; Xi et al., 2026
High-acuity optics	Raptor, ^*^Anableps^*^	Premium IOLs; wavefront correction; implant design	Clinical (optics); Theoretical (biological acuity gain)	Mitkus et al., 2017a; González-Martín-Moro et al., 2017; Mitkus et al., 2017b; Zueva et al., 2014; Potier, 2020; Potier et al., 2017; Schwab, 2002; Perez et al., 2017; Owens et al., 2009; Duan and Xu, 2022
Polarization vision	Mantis shrimp, cephalopod, insect	Bio-inspired polarization sensors	Preclinical	Liu et al., 2022; Dupeyroux et al., 2019; Liu et al., 2023; Miyata et al., 2020
Neural processing/coding	Insect motion circuits, mantis shrimp, snake	Prosthetic stimulation strategies; AI image preprocessing	Clinical (prostheses); Preclinical (AI coding)	Homberg et al., 2011; el Jundi et al., 2014
Gene therapy	Native gene function	Gene augmentation (Luxturna)	Clinical (approved)	Russell et al., 2017
Optogenetic restoration	Algal channelrhodopsin	AAV-delivered channelrhodopsin + goggles	Clinical	Sahel et al., 2021; Mohanty et al., 2025; Fong et al., 2025; Poboży et al., 2025; Bansal et al., 2021; Alekseev et al., 2025; Ganjawala et al., 2019; Lu et al., 2024; Sakai et al., 2022
Retinal prosthesis	Photoreceptor photovoltaic principle	Subretinal (PRIMA), epiretinal (Argus II) implants	Clinical,	Palanker et al., 2020; Delyfer et al., 2020; Bloch and da Cruz, 2019; Finn et al., 2018; Muqit et al., 2024; Bhuckory et al., 2025
Biomimetic sensors	Compound eye, pit organ, polarization	Multispectral, IR, polarization imagers	Preclinical to Proof-of-concept	Zhang et al., 2021b; Kogos et al., 2020; Ding et al., 2022; Mu et al., 2025

Finally, we define three terms that are used inconsistently in the literature and that we keep distinct throughout this review. **Restoration** is the recovery of a visual function that has been lost to disease or injury, back toward the species-typical human range. **Extension** is the expansion of a visual capacity beyond the native human range but within a range demonstrated by another species (for example, near-infrared perception). **Enhancement** is the improvement of a native capacity beyond its normal human maximum. Most of this review concerns restoration; extension and enhancement are addressed separately in the Discussion. We restrict ourselves to published, peer-reviewed evidence.

## 2 Retinal and eye regeneration: rebuilding the neural retina

The most direct way to restore vision loss due to retinal degeneration is to replace the lost cells. Humans cannot do this endogenously, but several vertebrate and invertebrate species can (Figure 1), and their mechanisms define the approaches for regenerative ophthalmology. All vertebrate species have the same basic retinal cell types: photoreceptors (PRs), bipolar cells (BPCs), retinal ganglion cells (RGCs), horizontal cells (HCs), amacrine cells (ACs), Müller glia (MG), and retinal pigment epithelium (RPE), yet only some species deploy them for repair. Understanding why reveals the molecular levers available for therapeutic intervention in humans.

**Figure 1:**
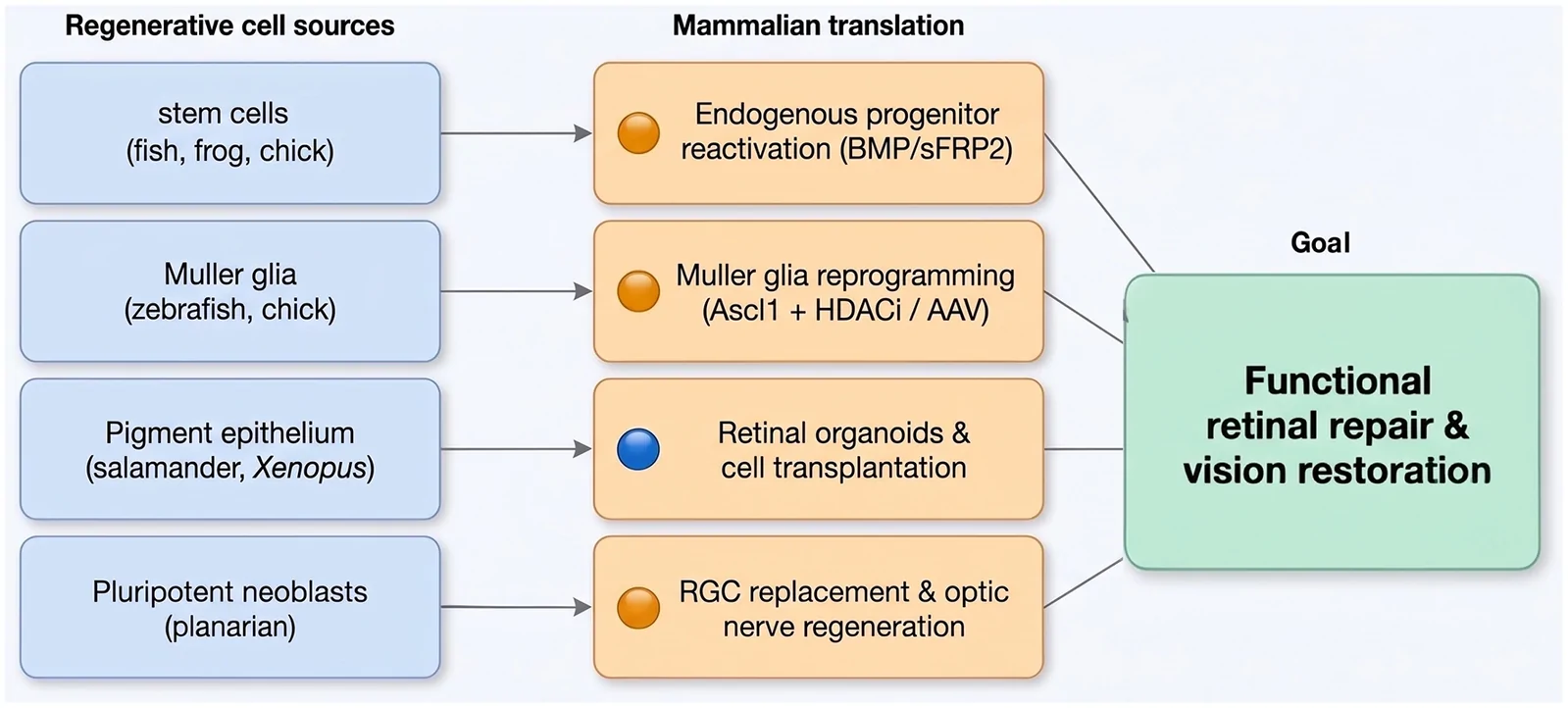
Retinal regeneration across species and translation to human therapy. Comparison of regenerative cell sources ciliary marginal zone stem cells, Müller glia, pigment epithelium transdifferentiation, and pluripotent stem cells across zebrafish, salamander, chick, Xenopus, planarian, and mouse, converging on human translational strategies: endogenous reprogramming, endogenous stem/progenitor reactivation, cell and organoid transplantation, and gene/optogenetic therapy. Yellow, preclinical; Blue, clinical stage.

Regenerative competence is not a single mechanism, but is distributed across several cellular sources whose relative contributions vary by species: a persistent ciliary marginal zone (CMZ) of retinal stem/progenitor cells, Müller glia that dedifferentiate into progenitors, pigment epithelial cells that transdifferentiate, and pluripotent stem cells. Regenerating species typically combine more than one of these sources, whereas mammals have lost or silenced nearly all of them.

### 2.1 Zebrafish: Müller glia as an endogenous retinal stem cell

Zebrafish (*Danio rerio*) regenerate a fully functional retina after widespread photoreceptor ablation, retinal puncture, or chemical lesion, restoring both structure and visually guided behavior within weeks (Powell et al., 2016; Celotto et al., 2023). The principal cellular engine of this process is the Müller glial cell, the same radial glial cell that, in mammals, responds to injury with reactive gliosis. Upon injury, zebrafish Müller glia dedifferentiate, re-enter the cell cycle, and generate multipotent progenitors that migrate to all retinal layers and differentiate into the appropriate neuronal types, with a proliferative bias toward the ablated layer (Powell et al., 2016). Single-cell transcriptomics has resolved the trajectory: injury drives Müller glia into a hybrid glial/progenitor state from which neurogenic clusters emerge (Figure 2), regenerating ganglion cells first and bipolar cells last (Celotto et al., 2023; Lyu et al., 2023).

**Figure 2:**
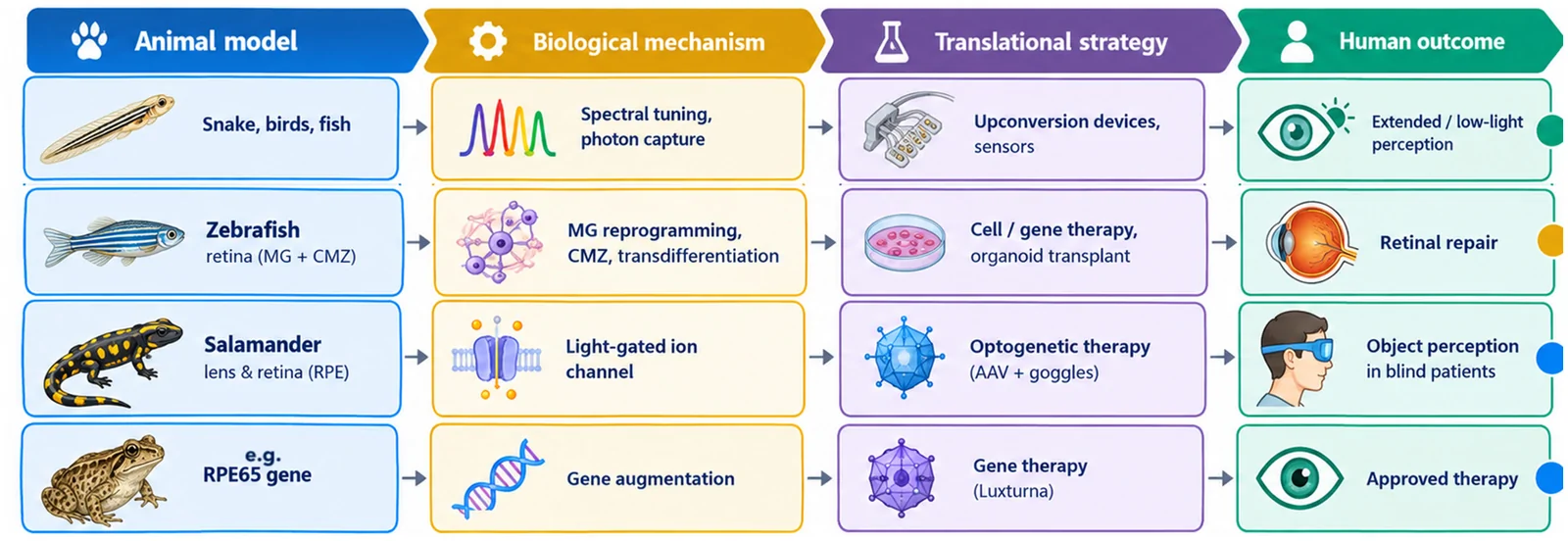
Four-stage flow from animal models and their visual specializations, through biological mechanisms and translational strategies, to human outcomes in vision restoration. Gene therapy, optogenetics, prostheses, cell therapy and vision extension (spectral extension, improved low-light/acuity), annotated with evidence grades. Green, proof of concept; Yellow, preclinical; Blue, clinical stage.

The molecular control of this program is increasingly well mapped, and the proneural transcription factor *Ascl1a* sits near its apex. *Ascl1a* is rapidly and specifically upregulated in zebrafish Müller glia after injury and is required for their dedifferentiation and proliferation, acting in part through a *Lin-28*—*let-7* axis that reactivates regenerative gene expression (Ramachandran et al., 2010; Fausett and Goldman, 2006). Transforming growth factor-β (TGF-β) signaling plays a biphasic role, first driving Müller glia reprogramming and later restraining progenitor proliferation (Sharma et al., 2020). Yes-associated protein (Yap) is required for the reprogramming of Müller glia into proliferating progenitors (Lourenço et al., 2021). Inflammatory signals from microglia and dying neurons coordinate the entire multi-step process, regulating Müller glia quiescence, reprogramming, proliferation, and the survival and differentiation of regenerated neurons (Nagashima and Hitchcock, 2021). Bromodomain-containing transcriptional regulators and injury-specific gene regulatory networks further distinguish regenerative neurogenesis from developmental neurogenesis, indicating that regeneration is not simply a recapitulation of development (Lyu et al., 2023; Lee et al., 2023). This last point is critical for translation: it demonstrates that the regenerative program is a distinct, inducible state, separable from embryogenesis, and therefore a legitimate target for therapeutic activation in a mammalian retina. The central role of *Ascl1a* in zebrafish is directly relevant to the mammalian reprogramming strategies discussed in Section 2.6, where its mammalian ortholog *Ascl1* is the most widely used reprogramming factor.

### 2.2 The ciliary marginal zone: a persistent retinal stem cell niche

In fish, amphibians, and to a limited extent birds, the retina also grows throughout life from a ciliary marginal zone (CMZ), which is a ring of proliferative retinal stem and progenitor cells at the retinal periphery, adjacent to the ciliary body (Fischer et al., 2013; Miles and Tropepe, 2021). Clonal and time-lapse analyses in zebrafish show that the CMZ contains bona fide, slowly cycling retinal stem cells that continuously add new retinal neurons of all types to the growing eye, linking embryonic and postembryonic retinal growth (Wan et al., 2016). In teleost fish, this marginal stem cell population coexists with a dedicated rod-lineage precursor and with injury-responsive Müller glia, so that lifelong growth and injury-induced regeneration draw on overlapping but distinct progenitor pools (Otteson and Hitchcock, 2003). In *Xenopus*, the CMZ is the principal source of the regenerated retina after complete retinal removal (Miyake and Araki, 2014; Xue and Harris, 2012; Ail and Perron, 2017).

The relative contributions of the CMZ and of Müller glia differ across species and injury types. In zebrafish, the CMZ drives lifelong growth, whereas acute regeneration of the central retina after a focal lesion is driven mainly by Müller glia; the two systems are spatially and molecularly distinct but share downstream effectors (Otteson and Hitchcock, 2003; Miles and Tropepe, 2021). This division of labor matters for translation, because it means that “regenerative competence” is not a single switch but a set of partially independent programs. Critically, mammals largely lack a functional CMZ: the mammalian retinal margin ceases to add neurons after early postnatal life, and no robust, self-renewing CMZ-like stem cell population persists in the adult mammalian eye (Fischer et al., 2013; Frøen et al., 2013). The evolutionary loss of robust CMZ activity is thus one of the clearest biological differences between regenerating vertebrates and mammals, and it raises the question addressed in Section 2.5 of whether a CMZ-like stem cell state can be reactivated or engineered in the mammalian eye.

### 2.3 Salamanders: transdifferentiation of pigment epithelium

Urodele amphibians (newts and salamanders) regenerate the lens and, in some species, the neural retina, through a fundamentally different mechanism: transdifferentiation of pigmented epithelial cells which is the process where specialized pigment cells in the eye change into a completely different type of cells. In Wolffian lens regeneration, dorsal iris pigment epithelial cells re-enter the cell cycle, dedifferentiate, and redifferentiate into crystallin-expressing lens fibers, a process driven by signals from the neural retina and characterized by graded dorsal-iris gene expression (including *Tbx5, Fgf10*, and *Vax2*; Tsonis et al., 2004; Sousounis et al., 2013; Henry and Hamilton, 2018). The axolotl regenerates its lens from either the dorsal or ventral iris during a restricted developmental window, demonstrating greater plasticity than the classic newt model (Suetsugu-Maki et al., 2012). Aging slows but does not abolish the process, delaying cell-cycle re-entry and extracellular matrix clearance (Tsissios et al., 2023).

Importantly, the evidence for pigment-epithelial transdifferentiation extends beyond the lens to the neural retina, though with important caveats. In the adult newt, surgical removal of the neural retina triggers retinal pigment epithelial (RPE) cells to proliferate and generate a new neural retina; recent work shows that these RPE cells are reprogrammed into a unique multipotent state resembling the early optic vesicle, from which both the neural retina and the RPE are rebuilt with correct polarity (Islam et al., 2014; Grigoryan and Markitantova, 2016). This process depends on tissue interactions with the underlying choroid and on the transcription factor *Pax6*, whose knockdown diverts reprogrammed RPE cells into a non-neural, myofibroblast-like fate (Mitsuda et al., 2005; Casco-Robles et al., 2016). RPE-to-neural-retina transdifferentiation is also documented in post-metamorphic *Xenopus* and, under experimental conditions, in embryonic chick, indicating that pigment epithelial plasticity is distributed across vertebrates (Yoshii et al., 2007; Araki, 2007). These findings carry a limitation relevant to translation: the cell-type specification of pigment-epithelial progeny is context-dependent and can be incomplete or misdirected (for example, toward lens fibers vs. neurons, or toward non-neural fates when key transcription factors are absent), reflecting the distinct developmental origins of the iris, RPE, and neural retina. In salamander terminally differentiated ocular cells can be coaxed into a new identity. The fidelity and completeness of this lineage conversion depend on chromatin remodeling, permissive epigenetic landscapes, and precise morphogen gradients (e.g., FGF and Wnt signaling; Araki, 2007) to effectively rewire the core gene regulatory networks (GRNs) governing target cell identity.

### 2.4 Frogs, chicks, and fish: growth-factor-driven repair and the regenerative spectrum

*Xenopus* tadpoles regenerate the entire retina after complete retinal removal, sourced from stem/progenitor cells in the CMZ (Miyake and Araki, 2014; Xue and Harris, 2012; Ail and Perron, 2017). The embryonic chick provides an intermediate model: its Müller glia can be driven to form Müller glia-derived progenitor cells (MGPCs) by fibroblast growth factor (FGF) and mitogen-activated protein kinase (MAPK) signaling, with bone morphogenetic protein (BMP)/*Smad1/5/8* promoting and TGF-β/Smad2/3 restraining progenitor formation (Fischer et al., 2008; Todd et al., 2017). Nuclear Factor I (*NfI*) suppress the neurogenic competence of these progenitors, and midkine and protein phosphatases fine-tune the balance between glial reactivity and neurogenesis (El-Hodiri et al., 2021; Campbell et al., 2021; Kelly et al., 2024). Midkine (*Mdk*) is a small, secreted heparin-binding growth factor involved in development, cell survival, proliferation, migration, and tissue repair. The chick also retains a residual CMZ-like zone and can mount limited RPE transdifferentiation under *Fgf* stimulation, placing it partway between the fully regenerative fish and the non-regenerative mammal (Fischer et al., 2013; Araki, 2007). These non-mammalian models collectively identify the signaling control, that determine whether Müller glia become a regenerative progenitor or a gliotic scar, and they map a regenerative spectrum through *FGF/MAPK, BMP, TGF-*β*, Notch, Hippo/Yap* signals.

### 2.5 Planarians: whole-eye regeneration from pluripotent stem cells

Planarians demonstrate *de novo* eye organogenesis from adult pluripotent stem cells (neoblasts), rebuilding both photoreceptors and the optic cup through a hierarchical gene regulatory network governed by *ovo, six1/2*, and *eya* (Wagner et al., 2011; Reddien, 2012). Notably, this regenerative program operates independently of *Pax6*, which is essential for eye development in vertebrates and Drosophila. This evolutionary divergence highlights the substantial translational distance between invertebrate rhabdomeric organogenesis and the functional replacement of human ciliary photoreceptors (Reddien, 2012; Klöppel et al., 2021).

Eye regeneration in planarian worms is driven by neoblasts that sense missing tissue identity and utilize lineage-restricted progenitors under the control of transcription factors such as *dlx* and *sp6-9* (Lapan and Reddien, 2011; LoCascio et al., 2017). Single-cell transcriptomics and RNAi screening have resolved a hierarchical, stepwise program of progenitor specification and differentiation into mature photoreceptors and optic cup cells (Scimone et al., 2026, 2025). A conserved core of *ovo, six1/2*, and *eya* specifies all eye progenitors, after which *otxA*, orthodenticle homeobox, a transcription factor involved in nervous-system and sensory development, segregates photoreceptor fate and *sp6-9*/*dlx* segregate pigment-cup fate (Scimone et al., 2025). RNAi of both planarian *Pax6* paralogs impairs neither eye regeneration nor maintenance, whereas *sine oculis, eya*, and *six-1* are essential (Pineda et al., 2002, 2000; Mannini et al., 2004).

### 2.6 Translating regeneration to mammals: potential reprogramming Müller glia

The central translational question is whether the mammalian retina, which shares the same cell types as regenerating species, can be induced to repair itself. The answer, from a decade of intensive work, is a qualified yes. Overexpression of the proneural transcription factor *Ascl1* (the mammalian ortholog of zebrafish *Ascl1a*) in adult mouse Müller glia, combined with a histone deacetylase inhibitor, stimulates the generation of functional retinal neurons after injury (Jorstad et al., 2017). Combining *Ascl1* with *Atoh1* broadens the repertoire of regenerated neuronal types (Todd et al., 2021), and targeted expression of developmental ganglion-cell transcription factors regenerates retinal ganglion-like cells (Todd et al., 2022). Adeno-associated virus (AAV)-mediated delivery of proneural factors reactivates the cell cycle in adult Müller glia and produces bipolar-, amacrine-, and ganglion-like neurons *in vivo* (Pavlou et al., 2025). The efficiency of mammalian reprogramming is constrained by *STAT* (Signal Transducer and Activator of Transcription) signaling, which limits *Ascl1* chromatin binding and neural regeneration, and by species-specific differences in *Notch, TGF-*β, and chromatin accessibility (Jorstad et al., 2020; Xiao et al., 2023).

Two features of the mammalian response are important for possible translation. First, the outcome depends on the injury model and experimental context: potential reprogramming efficiency and the spectrum of neuronal types generated differ between NMDA-induced excitotoxic injury, light damage, and genetic degeneration models, and between *in vivo* and explant preparations, so results from one paradigm do not automatically generalize (Lahne et al., 2020; Pavlou et al., 2025; Jorstad et al., 2020). Second, the therapeutic value of reprogramming depends on which cell type is generated: producing new photoreceptors or bipolar cells is useful for outer-retinal disease, whereas producing ganglion cells is necessary for glaucoma but far harder to achieve. Current *Ascl1*-based protocols generate predominantly inner-retinal neurons (amacrine- and bipolar-like cells) and few *bona fide*, fully integrated photoreceptors, which limits their present applicability to outer-retinal degeneration (Lahne et al., 2020; Todd et al., 2021, 2022). Mammalian Müller glia reprogramming is probably feasible, but not yet complete enough for clinical use; the priority is to close the efficiency gap by targeting epigenetic barriers and combinatorial transcription-factor codes identified in regenerating species (Lahne et al., 2020; Xiao et al., 2023).

### 2.7 Translating regeneration to mammals: possible reactivating endogenous retinal progenitor cells

Müller glia reprogramming is not the only possible route to endogenous repair. A complementary body of evidence might indicate that the adult mammalian eye retains quiescent retinal stem/progenitor populations whose regenerative activity is actively suppressed. The classic demonstration is that pigmented cells of the adult mammalian ciliary body (the pars plana/pars plicata epithelium, the anatomical remnant of the CMZ) can be isolated and induced to form self-renewing, multipotent neurospheres that express retinal progenitor markers such as *Sox2, Vsx2, Pax6, Rax, Sox9*, proliferation markers such as *Mki67, Pcna, Top2a*, and can differentiate into retinal neurons *in vitro* (Tropepe et al., 2000). Growth factor-responsive progenitors have also been identified in the postnatal mammalian retinal periphery (Zhao et al., 2005), and intraocular injection of growth factors might reactivate a quiescent proliferative population in the adult mammalian ciliary body *in vivo*, although the ciliary body environment is non-permissive for their migration and neurogenesis (Abdouh and Bernier, 2006). More recently, *in vivo* antagonism of the inhibitory signals *BMP* and the *Wnt* modulator *sFRP2* was shown to potentially stimulate quiescent ciliary epithelium stem cells in adult mammals to proliferate, migrate into the retina, and differentiate into mature retinal neurons, including photoreceptors and ganglion cells (Grise et al., 2021).

These findings are important because they reframe mammalian regenerative failure: it reflects not only the absence of regenerative cell populations, but also the presence of molecular mechanisms *BMP, Wnt/sFRP2, Notch*, and *Hippo* signaling, among others that actively restrict their activation. At the same time, the field is appropriately cautious: critical assessments note that adult human ciliary body epithelium has not been shown to contain a *bona fide*, self-renewing retinal stem cell population with robust *in vivo* neurogenic output, and the efficiency, retinal integration, and functional contribution of reactivated cells remain low (Frøen et al., 2013).

Endogenous stem/progenitor reactivation is best regarded as a promising but early-stage complement to Müller glia reprogramming, sharing the same central challenges of efficiency, maturation, and synaptic integration.

### 2.8 Retinal ganglion cell replacement and optic nerve regeneration

Most regenerative strategies target the outer retina, but diseases such as glaucoma, which is one of the leading causes of irreversible blindness, destroys the retinal ganglion cells (RGCs) and their axons. RGC replacement is a categorically harder problem than photoreceptor replacement, because a new RGC must not only survive and integrate into the retinal circuit but also extend an axon over a long distance (8 cm in humans), navigate to the correct brain targets, and re-establish retinotopic connections (Soucy et al., 2023; Zhang et al., 2021a). Stem cell-derived RGCs can now be generated at scale and transplanted into healthy and glaucomatous retinas, where a tiny fraction might survive, extend neurites, and integrate into the ganglion cell layer (Oswald et al., 2021; Agarwal et al., 2023). A key formidable barrier is the existing dense structural architecture of the retina and inner limiting membrane (ILM), which physically impedes donor RGC engraftment and requires adjustments in surgical transplantation procedures to deliver under ILM. Disrupting the ILM markedly improves donor cell survival, migration into the RGC layer, and axon extension toward the optic nerve head, but also causes some additional adverse events of the procedure itself (Aguzzi et al., 2026).

The greater obstacle, however, lies beyond the eye. Adult mammalian RGC axons normally fail to regenerate through the optic nerve, but this failure is now known to be reversible in principle: co-deletion of the growth-suppressing genes *PTEN* and *SOCS3* in adult RGCs drives robust, long-distance axon regeneration (Sun et al., 2011; Smith et al., 2009), and combining such genetic manipulation with chemorepulsive guidance cues and enhanced neuronal activity has enabled regenerating axons to reinnervate brain targets and partially restore simple visual behaviors and reflexive responses (de Lima et al., 2012; Zhang et al., 2025).

These studies establish that the adult mammalian visual pathway retains a latent capacity for target-specific reinnervation, but they also underscore the remaining hurdles: only a small fraction of 5% RGCs regenerate axons, retinotopic mapping is imprecise, myelination and synapse formation at central targets are inefficient, and no approach has yet restored patterned, high-acuity vision after optic nerve damage (Soucy et al., 2023; Liu et al., 2025). Autoimmune response further hinders the implementation of this approach. RGC replacement and optic nerve regeneration thus remain among the most formidable and most clinically important frontiers in vision restoration.

### 2.9 Retinal organoids and stem cell-based retinal replacement

Where endogenous repair is insufficient, transplantation of stem cell-derived retinal cells offers a complementary route, and the rise of retinal organoid technology has transformed this field. Human pluripotent stem cells (hPSCs), including embryonic stem cells and induced pluripotent stem cells (iPSCs), can be directed to self-organize into three-dimensional retinal organoids that recapitulate retinal development, producing laminated tissue containing photoreceptors, bipolar cells, RGCs, and Müller glia (Mandai, 2023; Gagliardi et al., 2019). Retinal organoids now serve three translational functions: they are a platform for disease modeling and drug screening; they are a scalable, renewable source of donor photoreceptors and other retinal neurons for transplantation; and they provide a developmental comparison point for understanding how regenerating species rebuild retinal tissue (Mandai, 2023).

Transplantation of organoid-derived photoreceptors has shown that purified human pluripotent stem cell-derived cone photoreceptors can restore visual function in advanced retinal degeneration models, providing preclinical validation for photoreceptor replacement (Ribeiro et al., 2021). Photoreceptor precursor transplantation can improve vision in degeneration models, in part through cytoplasmic material exchange between graft and host cells (Léveillard and Klipfel, 2019). Most significantly, the approach has entered the clinic: a first-in-human trial transplanting allogeneic iPSC-derived retinal organoid sheets into patients with advanced retinitis pigmentosa demonstrated graft survival and safety over 2 years, with structural evidence of photoreceptor rosette formation and, in some patients, improvements in light perception (Iwama et al., 2025; Zhang and Jin, 2024). In parallel, human embryonic stem cell-derived RPE patches have shown feasibility and safety in phase 1 studies for age-related macular degeneration, with visual acuity gains in some patients (da Cruz et al., 2018), and clinical-grade iPSC-derived RPE patches on biodegradable scaffolds rescue retinal degeneration in rodent and pig models (Sharma et al., 2019).

The persistent challenges are common across cell-replacement approaches: immune rejection, long-term graft survival, functional synaptic integration with host bipolar cells, the limited maturity of organoid-derived photoreceptors (which often remain in an outer-segment-poor, developmentally immature state), the absence of vasculature within organoids, and the need for robust, durable visual improvement (Zarbin, 2019; Liu et al., 2024; Mandai, 2023; Ye et al., 2025).

Regenerating species inform these efforts by revealing the extracellular matrix, inflammatory, and signaling environments that permit new neurons to integrate into a functioning retinal circuit environment that must be recreated or mimicked for transplanted cells to succeed in a degenerated human retina.

## 3 Spectral tuning: restoring and extending the visible band

The regenerative mechanisms discussed above focus on rebuilding or replacing damaged retinal tissue. However, restoration of vision is not limited to cellular regeneration. Across evolution, numerous species have addressed visual challenges by modifying how visual information is captured, processed, and interpreted. These adaptations provide an additional source of inspiration for therapeutic innovation. We consider mechanisms that restore or optimize visual function through spectral tuning, enhanced photon capture, optical specialization, and neural processing.

Human color vision is trichromatic and bounded by a lens that blocks ultraviolet (Cronin and Bok, 2016; Peng et al., 2024) and photoreceptors that do not extend into the infrared (Toomey et al., 2016; Hogg et al., 2011). Many species exceed both limits, and their adaptations are directly relevant to two restoration problems: recovering spectral function lost to disease or aging (for example, the loss of short-wavelength sensitivity after cataract or in blue-cone monochromacy; Cronin and Bok, 2016; Toomey et al., 2016), and designing bio-inspired devices that deliver spectral information the degenerated retina can no longer capture (Hogg et al., 2011). The recurring theme is that spectral function requires coordinated tuning of the opsin, the ocular media, and the neural circuitry which requires significant brain plasticity and a systems-level intervention (Figure 3).

**Figure 3:**
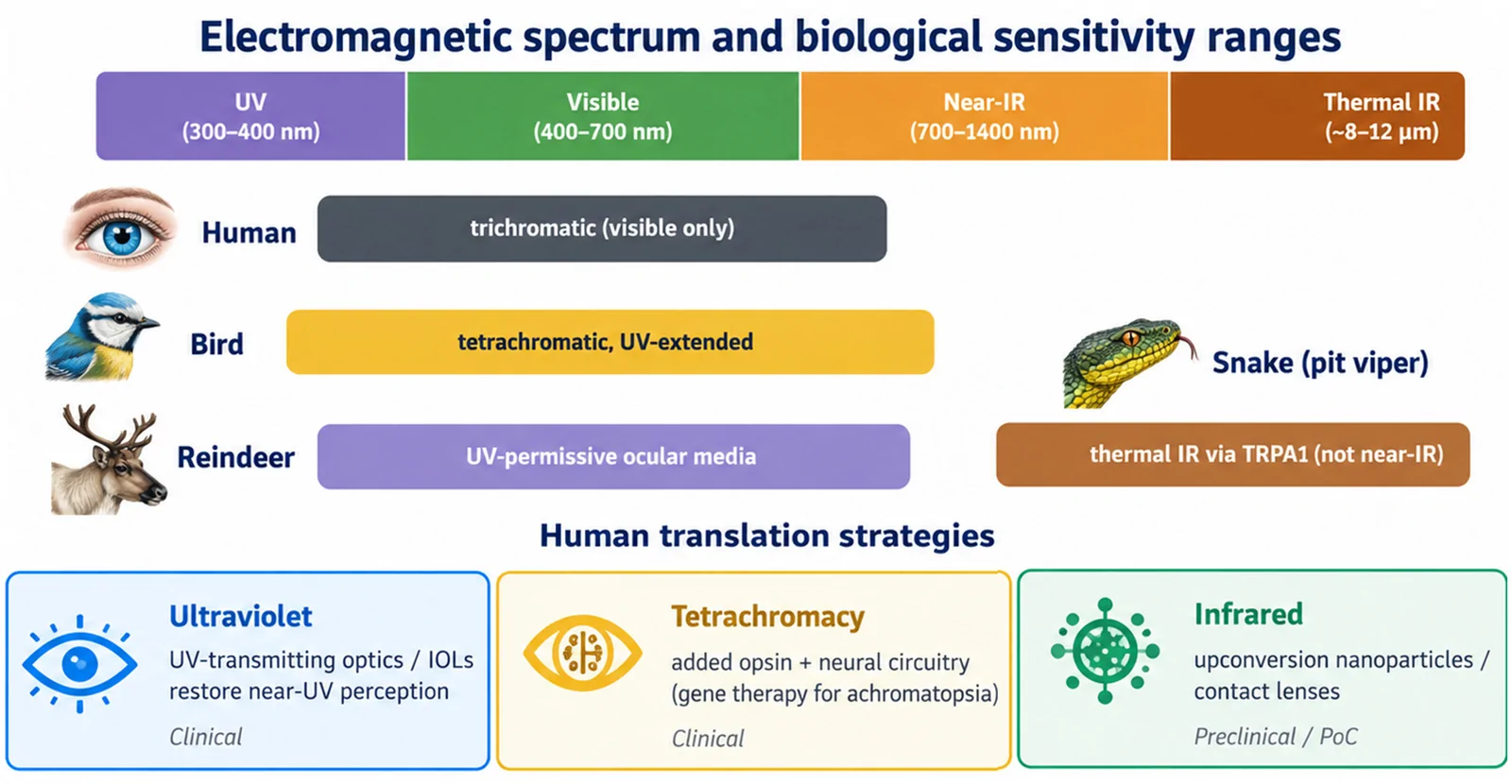
Expanding the visible spectrum: biological adaptations and human translation. **(Top)** wavelength spectrum with human (trichromatic), bird (tetrachromatic, UV-extended), and snake (thermal infrared) sensitivity. **(Bottom)** three strategies ultraviolet (optics/opsin), tetrachromacy (added opsin + neural circuitry), and infrared (upconversion nanoparticles).

### 3.1 Ultraviolet vision and the ocular media

Even though ultraviolet (UV) spectrum is damaging for the human retina, we observe that retinal UV sensitivity in the animal kingdom is widespread (Hogg et al., 2011; Tyler et al., 2014). Most birds, many fishes, numerous insects, and some mammals possess short-wavelength-sensitive (*Sws1*) opsins tuned to UV and ocular media that transmit it (Cronin and Bok, 2016; Peng et al., 2024). In birds, UV vision has evolved repeatedly, at least 8 shifts between UV-sensitive and violet-sensitive *Sws1* classes in passerines alone, and at least 14 shifts across all birds, often driven by single nucleotide substitutions in the *Sws1* opsin (Ödeen et al., 2011; Ödeen and Håstad, 2013). Ser86Phe or Ser86Ala substitution in *Sws1* opsin modify spectral wavelengths that can transition a pigment from violet to ultraviolet sensitivity which is observed in shorebirds and gulls (Ödeen and Håstad, 2013). Crucially, UV vision requires more than a UV opsin: the cornea and lens must transmit UV, and in birds the lens is the limiting factor, with ocular media transmittance tuned to the opsin class (Olsson et al., 2021; Lind et al., 2013). Birds further optimize UV color vision by complementary spectral tuning of the adjacent *Sws2* cone through carotenoid filters, achieving near-uniform spectral sampling across the UV–visible range (Toomey et al., 2016).

The reindeer illustrates how the same spectral extension can be achieved by a different route, and clarifies the underlying mechanism. Reindeer extend their visual range into the UV, an adaptation that enhances discrimination of UV-absorbing lichens, forage plants, and predators against UV-reflective snow during the dark Arctic winter (Hogg et al., 2011; Tyler et al., 2014; Dominy et al., 2023). Critically, this is not mediated by a novel or retuned opsin: reindeer retain a conventional mammalian *Sws1* cone opsin, and detailed physiological study has found no evidence of a specific boreal adaptation in their photoreceptors (Tyler et al., 2024). Instead, the extension is optical: the reindeer cornea and lens transmit far more UV than those of humans (roughly 15-fold greater transmission of short-wavelength light), so that UV reaches otherwise ordinary photoreceptors (Tyler et al., 2016; Hogg et al., 2011). The reindeer confirm that spectral range is set by the ocular media and by the opsin, so changing the optical filter alone can extend vision.

Humans are not blind to UV because of the photoreceptors. SWS cones can respond to near-UV, but the crystalline lens absorbs UV. Aphakic individuals (lacking a lens) and those with certain UV-transmitting intraocular lenses can perceive near-UV light, a phenomenon famously reported by the painter Claude Monet after cataract surgery (Cronin and Bok, 2016). This natural experiment demonstrates that the human visual system can process an extended short-wavelength signal when the optical filter is removed, and it defines a restoration strategy: engineering UV-transmitting optics to recover short-wavelength function while preserving retina from UV damage.

The translational relevance, however, requires robust benefit–risk analysis. The potential functional benefits of extended short-wavelength sensitivity in humans are modest and largely restricted to specific low-light or high-UV environments; set against these are well-documented risks, because chronic UV exposure is a cause of cataract, corneal damage, and retinal phototoxicity (Ivanov et al., 2018) as well as influence on circadian rhythms. Consequently, UV transmission is best regarded not as a general therapeutic objective but as a proof-of-principle that the human visual system can use an extended spectral band when the optical filter is removed. Any spectral intervention must be balanced against photoprotection (Ivanov et al., 2018).

### 3.2 Tetrachromacy and multiplied spectral channels

Most birds, reptiles, and fishes are tetrachromatic, possessing four cone classes and a correspondingly richer color space (Toomey et al., 2016; Ödeen et al., 2011). Their ability to perceive 80 million colors compared to human ability to perceive 1 million colors sets the stage for potential expansion. The mantis shrimp pushes this to an extreme, with up to 16 functional photoreceptor classes spanning UV, visible, and polarization channels, tuned by filtering pigments (Cronin et al., 2014). Remarkably, mantis shrimp color vision does not rely on fine-grained opponent comparison across these channels; instead, it uses a temporal, scanning-based recognition system that trades discrimination precision for speed (Thoen et al., 2014; Wang and Marshall, 2024). So, more spectral channels do not require proportionally more neural processing if the system is organized for recognition rather than discrimination.

Humans may harbor latent tetrachromacy. Because the red (*OPN1LW*) and green (*OPN1MW*) opsin genes are X-linked and highly polymorphic, female carriers of anomalous trichromacy can express four distinct cone opsins. However, most such carriers do not exhibit four-dimensional color vision with discrimination of dozens of millions of colors, likely because the cortex does not wire the extra channel into a functional opponent dimension (Jordan et al., 2010). The gap between retinal genotype and perceptual phenotype in human tetrachromacy is a central caution for any spectral intervention: adding a photoreceptor channel may be not sufficient without the neural circuitry to use it.

For restoration, the practical implication is that recovering lost spectral function requires attention to the neural readout, not just the opsin. This is illustrated by gene therapy for achromatopsia, a channelopathy of cone photoreceptors that abolishes color vision and acuity. Clinical trials delivering AAV-encoded *CNGA3* or *CNGB3* to the cone-rich macula have established safety and reported functional signals: subretinal AAV8.CNGA3 improved visual acuity and contrast sensitivity in adult patients (Fischer et al., 2020), with functional gains persisting at 2 and 3 years in follow-up of a phase I trial (Reichel et al., 2021), and first-in-human AAV8-hCARp.hCNGB3 was generally safe and well-tolerated with variable, generally modest improvements in cone-related measures (Michaelides et al., 2023). Treated children showed evidence of reactivated cone-mediated cortical pathways, revealing a degree of neural plasticity in the developing human visual system that adults may not share (Farahbakhsh et al., 2022). These results confirm that restoring a spectral function is clinically feasible, and the age-dependent plasticity of the neural readout is a major determinant of the functional benefit.

### 3.3 Infrared detection

Infrared (IR) detection has evolved independently in several lineages through non-opsin mechanisms. Pit vipers, boas, and pythons detect infrared radiation via warmth-activated TRPA1 ion channels in the pit organ, generating a thermal image of prey and predators (Gracheva et al., 2010; Panzano et al., 2010). Comparative genomics reveals convergent molecular evolution of *Trpa1* across these lineages, with parallel amino acid substitutions proposed to tune thermal sensitivity (Yokoyama et al., 2010; Geng et al., 2011). The vampire bat uses a related *Trpv1*-based mechanism in its nose leaf. True photonic IR vision in animals is thermal, not opsin-based.

For human application, the leading strategies are bio-inspired engineering, not gene transfer of a thermal channel, and the nature of the materials matters for their translational prospects. The injectable “retinal nanoantennae” were tested in mice, they are core–shell up conversion nanoparticles (UCNPs) typically a lanthanide-doped nanocrystal (for example, β-NaYF4 co-doped with ytterbium and erbium/thulium) coated with an inert shell and surface-functionalized (for example, with a photoreceptor-binding peptide) so that they anchor near the outer segments (Ma et al., 2019). These particles act as physical transducers that absorb two or more low-energy near-infrared photons and emit a single higher-energy visible photon (anti-Stokes upconversion), thereby converting NIR light into a signal the existing photoreceptors can detect without altering the opsin itself (Ma et al., 2019). In mice, subretinally injected UCNPs enabled perception and discrimination of NIR patterns (Ma et al., 2019). The same principle has been extended to humans non-invasively: wearable contact lenses doped with UCNPs enable human perception of near-infrared spatiotemporal patterns and even NIR “color” (Cai et al., 2019; Do, 2025). Ultralow-threshold upconversion designs and microsphere-mediated photofield concentration are improving sensitivity toward biologically relevant light levels (Wang et al., 2025a).

Two translational caveats deserve emphasis. First, the lanthanide-doped nanocrystals used in most UCNPs are not readily biodegradable, and their long-term retinal retention, clearance, and potential toxicity are not yet fully characterized (Ma et al., 2019; Gnach et al., 2015). Second, the wearable contact-lens format sidesteps this concern by placing the upconverting material outside the body, at the cost of lower sensitivity and the need for an external device (Cai et al., 2019; Do, 2025). These approaches effectively graft a new spectral band onto the existing human visual system without altering its genetics, and they illustrate a general principle: when a biological adaptation is hard to transplant, a physical one can sometimes deliver the same information through the native channel. For patients with advanced retinal degeneration, such devices could in principle deliver visual information through surviving retinal pathways. Wrapping up this section for multiplied spectral channels restoration, infrared detection and physical extension of human sensorial abilities one can refer to a living example, Neil Harbisson, the color-blind achromatopsia artist who uses an antenna implanted in his skull to perceive colors as sound calling it “eyeborg” technology (Farahbakhsh et al., 2022).

## 4 Vision in dim light: restoring scotopic function

Nocturnal and deep-sea species have evolved retinas that sacrifice acuity and color for extreme photon capture. Their adaptations are directly relevant to restoring scotopic (low-light) vision, which is among the first functions lost in retinitis pigmentosa and other rod-cone dystrophies, and to designing low-light sensors and prosthetic vision systems. The unifying principle is the sensitivity–acuity trade-off: every adaptation that increases photon capture or signal summation does so at the cost of spatial or temporal resolution.

### 4.1 Nocturnal birds and mammals

Owls are the archetypal low-light specialists, their retinas are rod-dominated. In the great horned owl, rods outnumber cones by roughly 30:1 and their visual system is optimized for scotopic acuity and contrast sensitivity at the expense of photopic resolution (Braekevelt, 1993; Orlowski et al., 2012). Genomic and transcriptomic studies in owls show strong positive selection of targeted genes underlying rod-mediated dim-light vision including *Pde6b, Cnga1, Cngb1, Sag*, and *Slc24a1*, whereas diurnal raptors show selection in cone phototransduction and UV-protection pathways, including *Cngb3, Xrcc5, Primpol, Mdm2*, and *Sirt1* (Wu et al., 2016; Borges et al., 2019). The barn owl's visual system is a clear demonstration of the sensitivity–acuity trade-off: it maximizes acuity under dark adaptation while accepting reduced resolution in bright light (Orlowski et al., 2012). Nocturnal primates such as tarsiers and owl monkeys, and nocturnal mammals such as cats, share a suite of adaptations: high rod density, a reflective tapetum lucidum that gives photoreceptors a second chance to capture photons, large pupils, and spatial and temporal summation in the retinal circuitry (Vee et al., 2022; Schwab et al., 2002). Among genes upregulated in animals with the tapetum lucidum there are riboflavin transport related genes *Slc52a2, Slc52a3, Slc25a19*, zinc transport related genes *Slc39a8, Slc39a14, Slc30a1, Slc30a3*, riboflavin kinase gene *Rfk*, taurine biosynthesis and transporter genes *Csad* and *Slc6a6* and taurine metabolism gene *Gadl1* (Schwab et al., 2002).

### 4.2 Deep-sea fishes: extreme photon capture

Deep-sea teleosts operate in the dimmest visual environment on Earth and display the most extreme low-light adaptations (de Busserolles et al., 2020). Many have pure rod retinas with high rod convergence onto ganglion cells, reflective tapeta, and large, often tubular eyes. The silver spinyfin (*Diretmus argenteus*) has multiplied its rod opsin genes 38 times, an unprecedented expansion that allows a single rod system to cover both the residual daylight and the bioluminescence spectrum, effectively adding spectral channels to scotopic vision (Musilová et al., 2018). Larvae carry up to seven *Rho* paralogs and adult individuals carry up to 14 *Rho* paralogs. Lanternfishes (*Myctophidae, Benthosema glaciale*) show diverse photoreceptor sizes, densities, and distributions tuned to downwelling light or bioluminescence, with depth and nocturnality predicting retinal design (de Busserolles et al., 2014; de Busserolles and Justin Marshall, 2017). Some deep-sea fish larvae even develop hybrid photoreceptors combining rod-like morphology with cone-like molecular machinery, revealing an alternative developmental trajectory for dim-light vision (Fogg et al., 2026). Rhodopsin itself is exquisitely tuned across species for phototransduction at different light levels, providing a molecular template for engineering sensitivity (Hofmann and Lamb, 2022).

### 4.3 Translating low-light adaptation

The translational value of these adaptations is threefold. First, they define the biophysical limits of photon capture and the circuit-level trade-offs (convergence, summation, tapetum) that maximize sensitivity. These parameters can be mimicked in image sensors and prosthetic vision. Second, they identify molecular targets: tuning rhodopsin kinetics or rod density is a plausible route to enhancing residual scotopic function in partial retinal degeneration, though this remains to be demonstrated. Third, they inspire device design, from owl-inspired neuromorphic sensors that detect ultraweak light in dim environments to low-light image intensifiers (Xi et al., 2026).

Low-light adaptation is most plausibly an intervention under specific, well-defined conditions. The clearest indication is residual scotopic function: in retinitis pigmentosa and related rod-cone dystrophies, night blindness and loss of dim-light vision are often the earliest and most disabling symptoms, appearing while cones and central acuity are still preserved. In this setting, even a modest gain in photon capture or signal-to-noise ratio can meaningfully extend the range of usable light levels paralleled with personal light sources used by such people to help them navigate through dim environments.

The preclinical devices inspired by these adaptations work by decoupling light detection from the damaged photoreceptors: owl-inspired neuromorphic sensors use event-based, high-gain photodetection with on-chip noise suppression to register sparse photons in dim environments (Xi et al., 2026), and low-light image intensifiers amplify the available photon flux and present a brightened image to the surviving retina through a wearable display. In both cases the device performs the photon capture and amplification externally and delivers the result through the patient's remaining retinal pathways.

Beyond devices, related principles are being explored through biological interventions in the mammalian retina. These include pharmacological approaches that modulate phototransduction gain or accelerate rhodopsin regeneration to improve rod sensitivity, gene-therapy strategies that preserve or rescue rods to maintain the scotopic range (Russell et al., 2017), and optogenetic approaches that render surviving inner-retinal neurons light-sensitive with high-gain opsins (Sahel et al., 2021; Hofmann and Lamb, 2022; Xi et al., 2026). The honest constraint is that human scotopic vision is limited not only by photoreceptors but by the optical quality of the eye and the noise floor of the retinal circuitry; nature's adaptations address all three, and so must any human intervention. A tapetum, for example, would be difficult to add to the human eye, but its photon recycling function can be approximated in a sensor.

## 5 High acuity and optical specializations: resolution limits for prosthetic vision

At the opposite end of the sensitivity–acuity trade-off, diurnal raptors and several other lineages have evolved visual systems with spatial resolution and optical flexibility far exceeding the human eye. These systems reveal that acuity is a systems property, emerging from the co-optimization of optics, photoreceptor density, and neural sampling. This principle directly constrains the design of retinal prostheses and optogenetic therapies along with its software design, which must match the eye's optical and neural limits to deliver useful resolution.

### 5.1 Raptor acuity and the double fovea

Diurnal raptors have the highest visual acuity in the animal kingdom, with large eagles and falcons resolving detail at roughly twice human acuity (Mitkus et al., 2017a; González-Martín-Moro et al., 2017). This performance rests on a tubular eye shape, a large pupil, and an extremely high density of photoreceptors in the fovea, molecular adaptations involve cone phototransduction genes upregulation, such as *Cngb3* and photoreceptor-maintenance genes upregulation including *Ccdc66, Rpgr*, and *Nxnl2*. In the central fovea, rods and, in several species, double cones are largely excluded, allowing densely packed single cones to support high-resolution tetrachromatic vision (Mitkus et al., 2017b; Zueva et al., 2014). Many pursuit-hunting raptors possess two foveae per eye: a deep central fovea for lateral, high-resolution viewing and a shallower temporal fovea that supports frontal, binocular viewing during prey pursuit (González-Martín-Moro et al., 2017; Potier, 2020). Eye size is a strong predictor of foveal depth and thus potential acuity, linking the optical and neural components of resolution (Potier et al., 2017). Müller cells in the bird fovea act as optical fibers, channeling light to individual cones and effectively increasing the sampling density of the photoreceptor mosaic. Müller cells are specialized alongside the raptor fovea to sustain its exceptionally dense photoreceptor population, with enhanced pathways for glutamate recycling (*Glul, Slc1a3*), potassium buffering (*Kcnj10*), retinoid metabolism (*Rlbp1*), metabolic coupling (*Slc16a1, Ldha*), and antioxidant protection (*Gpx1, Prdx6, Sod1*; Zueva et al., 2014).

### 5.2 Dual optics and independent eyes

The four-eyed fish Anableps anableps has evolved a different solution to a divided visual world: developmental remodeling produces paired dorsal and ventral pupils and corneal surfaces, while the retina becomes molecularly specialized for the two optical environments. The aquatic-facing dorsal retina preferentially expresses the long-wavelength-sensitive opsin *lws*, whereas the aerial-facing ventral retina expresses the medium-wavelength-sensitive *rh2-1*, superimposed on shared expression of *sws1, sws2b, rh2-2*, and *rh1* (Schwab, 2002; Perez et al., 2017). The ventral retina, which views the aerial field, is enriched in bipolar and ganglion cells with a correspondingly enlarged tectal representation, and the species expresses at least ten visual opsins, including a unique *Lws* locus, supporting its dual visual ecology (Perez et al., 2017; Owens et al., 2009).

In chameleons, independent visual worlds are supported by the convergence of specialized ocular development, cone-rich high-acuity retinas, and partially segregated visuomotor circuits. Eye-development pathways in chameleons involve upregulation of *Pax6, Rax, Sox2, Six3*, and *Vsx2* establishing retinal architecture, while cone phototransduction genes such as *Opn1sw, Opn1mw, Opn1lw, Gnat2, Pde6c*, and *Cngb3* support high-acuity vision. At the neural level, largely independent left- and right-eye premotor circuits can be decoupled during environmental scanning and coupled during prey tracking, allowing the animal to switch between monocular surveillance and coordinated binocular targeting. These systems demonstrate that the vertebrate eye can be optically and neurally partitioned to serve two visual tasks at once. This principle is directly relevant to the design of multifocal intraocular lenses and dual-mode prosthetic vision.

### 5.3 Translating high-acuity and optical design

The translational impact of high-acuity biology is primarily in optics and devices. Understanding how raptor eyes combine a large pupil, high cone density, and a deep fovea informs the design of premium intraocular lenses, wavefront-guided refractive corrections, and ocular implants that must match the eye's optical limits. The double fovea and *Anableps* dual-optics system inspire multifocal and dual-mode designs. Eagle-eye machine vision is an active area of artificial-intelligence research, aiming to replicate “knowing what is where by seeing” in real time (Duan and Xu, 2022).

The biological constraint is that human acuity is limited by the spacing of the foveal cone mosaic and the optics of the cornea and lens; nature shows that acuity gains come from co-optimizing optics, photoreceptor density, and neural sampling, not from any single change. Some humans achieve exceptionally high visual acuity through the extreme packing of cone photoreceptors in the foveola, where a dense, orderly cone mosaic supports resolving power reaching approximately 20/10 and, under optimized optical conditions, about 20/8 or better. Simply increasing the pixel count of a retinal implant does not proportionally increase acuity, the optics and the neural interface must keep pace.

## 6 Polarization and additional sensory channels

### 6.1 Biological polarization perception

Beyond intensity and wavelength, many animals extract polarization as a distinct visual channel. Polarization is the orientation of light's electric field. Mantis shrimps are the champions, with dedicated polarization-sensitive photoreceptors and the ability to detect circularly polarized light; they actively rotate their eyes to align receptors with the polarization angle and maximize contrast (Cronin et al., 2014; Daly et al., 2016). Cephalopods and many insects use polarization for navigation, prey detection, and communication. Humans retain only a rudimentary polarization sensitivity (Haidinger's brush), an entoptic phenomenon with no known ecological function.

At the molecular level, polarization vision emerges from the precise organization of photopigments and photoreceptor membranes. Dichroic opsins embedded in highly ordered microvilli (maintained partially by elevated function of *Actb, Actn*, and *Myo* genes) preferentially absorb light according to the orientation of its electric field, while orthogonally arranged photoreceptor channels provide complementary polarization signals that can be compared neurally by GABAergic and glutamatergic circuits. In mantis shrimps, this architecture is elaborated into specialized rhabdoms and optical filters that support both linear and circular polarization detection (upregulated genes include *NinaA, Arr1, Arr2, RdgC*), with torsional eye movements actively aligning photoreceptors to maximize polarization contrast. Humans have a much weaker version of this phenomenon. The Haidinger's brush arises because macular pigments and the ordered structure of the foveal Henle fiber layer preferentially absorb differently oriented polarized light. This is not a dedicated neural polarization channel comparable to that of insects or mantis shrimp.

The translational value of polarization vision for humans is arguable and is currently dominated by engineering. Bio-inspired polarization sensors based on artificial compound eyes provide accurate heading information for navigation (Liu et al., 2022; Dupeyroux et al., 2019). Stomatopod-inspired metasurfaces achieve wide-field, full-Stokes polarization imaging in ultra-thin form factors (Liu et al., 2023; Miyata et al., 2020). These devices demonstrate that a sensory channel humans barely possess can be implemented efficiently in silicon and meta-optics, with applications in machine vision, navigation, and biomedical imaging. Polarization also offers a practical advantage in medical imaging, where it can reveal tissue structure invisible to intensity-based imaging, suggesting a role for bio-inspired polarization sensors in ophthalmic diagnostics.

### 6.2 Bio-inspired polarization sensors and human translation

A natural question is how the nervous system of polarization-sensitive species processes this additional channel, and whether the human brain could do the same. In insects and cephalopods, polarization is not simply detected at the receptor but is processed by dedicated neural circuitry: polarization-opponent interneurons compare orthogonally oriented receptors, and in insects this information is funneled into a central-complex compass network that encodes heading relative to the skylight polarization pattern (Homberg et al., 2011; el Jundi et al., 2014). This is the same systems principle seen for spectral channels: an extra sensory dimension is useful only because dedicated downstream circuitry reads it out.

There is no evidence that the human retina or cortex contains polarization-opponent circuitry, and Haidinger's brush is thought to arise from the radial arrangement of macular pigments rather than from a functional polarization pathway. Consequently, adding biological polarization vision to humans would require not only a polarization-sensitive detector but also the *de novo* construction of opponent processing and readout circuitry, which is far more demanding undertaking than adding a spectral channel. The realistic path is wearable or implantable polarization sensors that transduce polarization into an intensity or color signal the existing visual system already understands.

## 7 Neural processing and the visual brain

### 7.1 Inner-retinal neural processing

Visual restoration requires more than converting light into electrical signals; it requires presenting information in a format that respects the retina's native microcircuitry. The outer retina splits visual input into parallel ON and OFF pathways via sign-preserving (AMPAR/kainate) and sign-inverting (mGluR6) glutamatergic synapses between photoreceptors and bipolar cells. Bipolar cell dendrites receive lateral inhibitory input from horizontal cells, establishing classic center-surround receptive fields that enhance spatial contrast and sharp edges before signals reach the ganglion cell layer. Amacrine cells further refine these signals by providing temporal gain control, direction selectivity, and crossover inhibition between ON and OFF channels.

This inner-retinal processing architecture dictates the design of both optogenetic and prosthetic interventions. Epiretinal implants and optogenetic therapies targeting retinal ganglion cells (RGCs) bypass this entire microcircuit, delivering artificial spatiotemporal spike trains directly to RGC axons. This complete bypass eliminates natural center-surround antagonism and dynamic range adaptation, contributing to the coarse, pixelated percepts reported in clinical trials. Conversely, subretinal photovoltaic implants (such as PRIMA) and bipolar-targeted optogenetics make use of surviving inner-retinal circuitry. By stimulating ON-bipolar cells, these approaches allow signals to pass through endogenous amacrine and ganglion cell networks, preserving element-level contrast processing, local gain control, and temporal sharpening.

### 7.2 Cortical visual processing, reorganization, and prosthetic vision

The ultimate recipient of restored visual input is the visual cortex. Primary visual cortex (V1) neurons are tuned to complex spatiotemporal features, including edge orientation, spatial frequency, ocular dominance, and binocular disparity. In adult patients with late-stage outer retinal degeneration, prolonged visual deprivation induces homeostatic plasticity, cross-modal cortical reorganization, and alterations in receptive field tuning.

Clinical optogenetic and prosthetic studies demonstrate that despite years or decades of blindness, adult V1 retains sufficient plasticity to process artificial, coarse signals and translate them into meaningful visual percepts. To bridge the gap between artificial stimulation and cortical expectation, next-generation vision restoration platforms incorporate neuromorphic and AI-driven image preprocessing.

Event-based camera sensors and deep-learning encoders compress ambient visual scenes into bio-inspired spike trains that mimic the natural firing rates and center-surround dynamics of RGC populations. By delivering biomimetic stimulation patterns to inner-retinal neurons or cortical surface arrays, these technologies reduce the perceptual learning burden on the patient's brain and maximize functional visual acuity.

The eye does not see; the brain does. Many animals achieve remarkable visual performance with modest optics by virtue of highly efficient neural processing. Insects such as flies and dragonflies detect and intercept prey with motion-detection circuits of legendary efficiency, computing direction-selective signals with a handful of neurons. The mantis shrimp's scanning color-recognition system shows that even a dozen spectral channels can be handled with limited neural hardware if the computation is organized appropriately (Thoen et al., 2014; Wang and Marshall, 2024). The snake pit organ's ability to extract milli-Kelvin thermal signals from noisy ion channels through a bifurcation in its spiking dynamics is a striking example of neural noise shaping (Graf and Machta, 2024).

For human vision restoration, the lesson is that the quality of a prosthetic or optogenetic percept depends as much on the neural code delivered to the brain as on the sensor. Retinal prostheses and optogenetic therapies must speak the retina's and cortex's language to produce useful vision. Appropriate spatiotemporal patterns, gain control, and adaptation are needed, which must be provided by the software of this wearable technology. Real-time closed-loop simulation for cortical visual prosthetics that dynamically adapts artificial-vision stimulation according to both visual input and moment-to-moment neural-state signals is being investigated at McGill University. Using a phosphene-based vision simulator, safety-constrained stimulation models, and publicly available EEG alpha-band data from 240 participants, the system adjusted stimulation frame by frame through a reward-based controller. The framework achieved 18–28 ms latency while maintaining modeled safety limits, and EEG-informed control improved perceptual stability, with more than 80% of frames converging toward consistent phosphene configurations. The system remained responsive to neural-state fluctuations and maintained stable percepts during rapidly changing, mobility-like visual scenes, suggesting that closed-loop incorporation of accessible neural signals could improve the stability and adaptability of artificial vision without compromising real-time performance (Mouawad, 2026).

Bio-inspired neural processing is therefore a critical, if sometimes underappreciated, component of the translational pipeline, informing stimulation strategies, AI-based image preprocessing for prostheses, and the design of cortical visual prostheses (Sarbout et al., 2025). As prosthetic and optogenetic systems mature, the bottleneck is shifting from hardware to neural coding: how to present information so that a brain that may have been blind for years can interpret it.

## 8 Bio-inspired technologies for vision restoration

The biological strategies reviewed above converge on a set of technologies that are now in clinical use or advanced development. This section evaluates the leading platforms and their biological inspiration, and Table 2 summarizes their translational status and evidence grade.

### 8.1 Gene therapy: replacing the missing function

The approval of voretigene neparvovec (Luxturna) for *RPE65*-mediated inherited retinal dystrophy marked the arrival of retinal gene therapy. In the pivotal phase 3 trial, treated patients showed a mean bilateral multi-luminance mobility test gain of 1.8 light levels vs. 0.2 in controls, with 65% of treated participants passing the lowest luminance level tested (Russell et al., 2017). Long-term follow-up shows sustained improvement in rod and cone function, though accelerated chorioretinal atrophy has emerged as a concern in a subset of treated eyes (Lorenz, 2025; Stingl et al., 2021). Gene therapy is the clearest example of restoring a biological function that nature evolved but a mutation broke; its success has opened the door to gene augmentation, gene editing, and optogenetic approaches for a growing list of inherited retinal diseases (Chiu et al., 2021), including the achromatopsia trials discussed in Section 3.2.

### 8.2 Optogenetic vision restoration: light-gated channels from microbes

Optogenetics restores light sensitivity to a degenerated retina by expressing light-gated ion channels in surviving retinal neurons, which were originally discovered in algae and other microbes.

The functional outcome of optogenetic vision restoration depends not only on opsin biophysics (spectral sensitivity, light sensitivity threshold, and temporal kinetics) but equally on circuit-level target cell specificity. Delivering light-gated opsins directly to retinal ganglion cells (RGCs) provides a direct route to project signals to the brain, but it completely bypasses the complex signal processing performed by inner-retinal networks. As a result, RGC-targeted expression eliminates natural center-surround receptive field antagonism, sign-inverting ON/OFF pathway segregation, lateral inhibition, and dynamic gain control, delivering an unrefined signal to the optic nerve.

Conversely, targeting surviving ON-bipolar cells (e.g., using cell-type-specific promoters such as mGluR6/GRM6) reinstates a major portion of the inner-retinal processing circuit. By capturing light upstream at the bipolar cell layer, the optogenetic signal flows through endogenous amacrine and ganglion cell microcircuits, partially restoring lateral inhibition, spatial edge enhancement, and temporal contrast processing before reaching visual cortex targets. Table 4 reflects these crucial target cell trade-offs, emphasizing that optimizing visual acuity and contrast sensitivity requires aligning opsin properties with circuit-level functional engineering.

**Table 4:** Opsins used or proposed for optogenetic vision restoration: properties, advantages, and limitations.

Opsin	Type/origin	Peak activation	Light sensitivity (irradiance threshold)	Kinetics	Target cells	Advantages	Limitations	References
ChR2	Channelr-hodopsin-2 (algal)	~470 nm (blue)	Low (high threshold)	Fast ON/OFF	Ganglion, bipolar	Well-characterized; fast temporal fidelity	Requires high-intensity blue light; phototoxicity risk; needs image-amplifying goggles	Poboży et al., 2025; Sakai et al., 2022
ChrimsonR	Red-shifted channel-rhodopsin	~590 nm (red)	Moderate	Fast	Ganglion	Red light = better tissue penetration, lower phototoxicity; first clinical success (with goggles)	Still needs bright light + goggles; limited acuity; no color	Sahel et al., 2021; Poboży et al., 2025
ReaChR	Red-shifted chimera	~590–610 nm	Moderate–high	Moderate	Ganglion, bipolar	Red-shifted; improved sensitivity over ChR2	Slower kinetics; still supplemental light	Poboży et al., 2025; Bansal et al., 2021
CoChR (engineered variants)	Chloromonas channel-rhodopsin	~470 nm	High	Fast	Ganglion, bipolar	Restores acuity & contrast under ambient light in blind mice (goggle-free)	Blue-green light; preclinical only	Ganjawala et al., 2019
ChRmine	Cation-conducting channelrhodopsin (CCR)	~520 nm	Very high (threshold ~10^4^ μW/mm^2^)	Slow closing	Ganglion, bipolar	Operates near ambient indoor light; high firing rates	Slow off-kinetics limit temporal resolution; preclinical	Fong et al., 2025; Bansal et al., 2021
ChroME	ChRmine-derived	~520 nm	Very high	Faster than ChRmine	Ganglion, bipolar	High sensitivity with improved kinetics	Preclinical; long-term stability unproven	Fong et al., 2025
ChReef	ChRmine-derived	~520 nm	Very high	Improved/sustained	Ganglion, bipolar	Reliable, sustained control at low light; validated in rodents & primates	Preclinical; recent	Alekseev et al., 2025
MCO1 (multi-characteristic opsin)	Synthetic chimera	Broad (blue–red)	High	Fast	Bipolar	Broad spectral sensitivity + fast kinetics in one construct; clinical shape discrimination & mobility gains	Small open-label trials; long-term durability unknown	Mohanty et al., 2025

In a landmark study, a blind patient with retinitis pigmentosa received an intravitreal AAV encoding the channelrhodopsin ChrimsonR and, with the aid of light-gated goggles, was able to perceive, locate, count, and touch objects, with object-related activity detected over the visual cortex (Sahel et al., 2021). Subsequent work has introduced synthetic multi-characteristic opsins delivered to bipolar cells, improving shape discrimination and mobility in small open-label trials (Mohanty et al., 2025), and next-generation high-sensitivity opsins such as ChRmine and ChroME that function at lower light intensities (Fong et al., 2025).

Because the choice of opsin is the central engineering decision in optogenetic therapy, and because different opsins embody different trade-offs, we summarize the leading candidates in Table 4. The key parameters are spectral sensitivity (peak activation wavelength), light sensitivity (the irradiance threshold for activation), temporal kinetics (how fast the channel opens and closes, which sets the achievable temporal resolution), and the target cell (ganglion vs. bipolar). First-generation channelrhodopsins such as ChR2 require high-intensity blue light and fast kinetics but poor sensitivity, necessitating image-amplifying goggles. Red-shifted opsins such as ChrimsonR and ReaChR reduce phototoxicity and improve tissue penetration but still require relatively bright light (Sahel et al., 2021; Poboży et al., 2025).

High-sensitivity opsins such as ChRmine and its derivatives (ChroME, ChReef) reach irradiance thresholds orders of magnitude lower, approaching ambient indoor light, at the cost of slower closing kinetics that can limit temporal resolution (Fong et al., 2025; Bansal et al., 2021; Alekseev et al., 2025). Engineered CoChR variants restore visual acuity and contrast sensitivity in blind mice under ambient light without goggles (Ganjawala et al., 2019), and synthetic multi-characteristic opsins (*MCO1*) aim to combine broad spectral sensitivity with fast kinetics in a single construct (Mohanty et al., 2025). No single opsin is optimal across all parameters; the field is actively matching opsin properties to disease stage, target cell, and the accompanying light-delivery hardware (Lu et al., 2024; Sakai et al., 2022).

Optogenetics is the most direct realization of “learning from nature”: a microbial light sensor, refined by evolution, is repurposed to replace a lost human one. The restored percept is of low spatial resolution. In the first clinical case it was sufficient to localize and count large high-contrast objects but far below normal acuity, because the opsin is expressed across many ganglion or bipolar cells without the eye's native point-by-point photoreceptor sampling (Sahel et al., 2021; Lu et al., 2024). Color perception is essentially absent, because a single opsin confers a single spectral sensitivity and cannot support opponent color comparison (Poboży et al., 2025). And long-term durability is unproven: transgene expression, immunogenicity, and the stability of the restored percept over years remain open questions, as does the need for repeated dosing (Lu et al., 2024; Sakai et al., 2022). The need for bright light and image-stimulating goggles, and the as-yet-limited acuity of the restored percept, are the focus of intense engineering.

### 8.3 Retinal prostheses: engineering the interface

Retinal prostheses bypass damaged photoreceptors by electrically stimulating surviving retinal neurons. The Argus II epiretinal system provided light perception and improved functional vision and mobility in patients with end-stage retinitis pigmentosa, with more than 70% of patients in a French post-approval study showing functional gains at 2 years. However, its clinical impact remained limited by relatively modest visual performance, the challenges of implanting and maintaining an epiretinal device in a severely degenerated retina, and device- and procedure-related complications, including conjunctival erosion, retinal detachment, hypotony, vitreous hemorrhage, and endophthalmitis. Although the system was discontinued commercially in 2019, the manufacturer attributed this decision primarily to commercial considerations and the limited size of the eligible patient population rather than to a single clinical failure (Delyfer et al., 2020; Bloch and da Cruz, 2019; Finn et al., 2018). The PRIMA wireless photovoltaic subretinal implant has restored central vision in patients with geographic atrophy, enabling reading with prosthetic visual acuity improving by up to eight ETDRS lines with zoom at 4 years, without compromising residual peripheral vision (Palanker et al., 2020; Muqit et al., 2024). Subretinal photovoltaic implants can be upgraded *in situ* to improve acuity (Bhuckory et al., 2025). Emerging approaches include tellurium nanowire retinal nanoprostheses that convert visible and near-infrared light into electrical signals and improve vision in blind animal models (Wang et al., 2025b). The trajectory is toward higher-density, wireless, and upgradeable devices that better match the retina's native resolution and code.

### 8.4 Biomimetic sensors and spectral devices

Bio-inspired engineering extends human vision without surgery. Curved, ommatidia-inspired compound-eye cameras achieve real-time multispectral imaging over an ultra-large field of view (Zhang et al., 2021b; Kogos et al., 2020). Ommatidium—is the basic visual unit of a compound eye, such as those of insects, crustaceans, and many other arthropods. Polarization cameras inspired by the mantis shrimp and insect compound eye deliver full-Stokes imaging in ultra-thin packages (Liu et al., 2023; Miyata et al., 2020). Snake-pit-organ-inspired uncooled infrared imagers detect body-temperature objects without external power (Ding et al., 2022), and CMOS-integrated upconverters achieve high-resolution short-wave infrared imaging (Mu et al., 2025). Wearable upconversion contact lenses extend human perception into the near-infrared (Cai et al., 2019; Do, 2025). These devices embody a pragmatic philosophy: when biological restoration of a visual function is difficult, build a bio-inspired interface that delivers the information through the eye's existing capabilities. They also blur the line between restoration and extension, since the same upconversion lens that could help a visually impaired person could also give a sighted person infrared vision.

## 9 Discussion

### 9.1 Synthesis: a mechanism-first map from biology to therapy

Viewed as a whole, the field reveals maturity of each path (Table 3). For **restoration**, the most mature paths are gene therapy (replacing a mutated gene), optogenetics (installing a microbial light sensor), and prostheses (engineering an electronic interface), with regenerative approaches advancing toward clinical feasibility: Müller glia reprogramming (preclinical), endogenous stem/progenitor reactivation (preclinical), and cell and organoid transplantation (clinical, early-phase). The biological specializations that have not yet yielded clinical therapies, such as spectral expansion, low-light adaptation, high-acuity optics, and polarization, nonetheless provide the mechanistic blueprints and bio-inspired devices that define the next generation of interventions (Figure 4). The mechanism-first organization makes explicit that the same biological insight, for example, opsin spectral tuning or Müller glia plasticity, can serve multiple translational goals, and that the limiting step is often not the sensor but the neural and optical context in which it operates.

**Figure 4:**
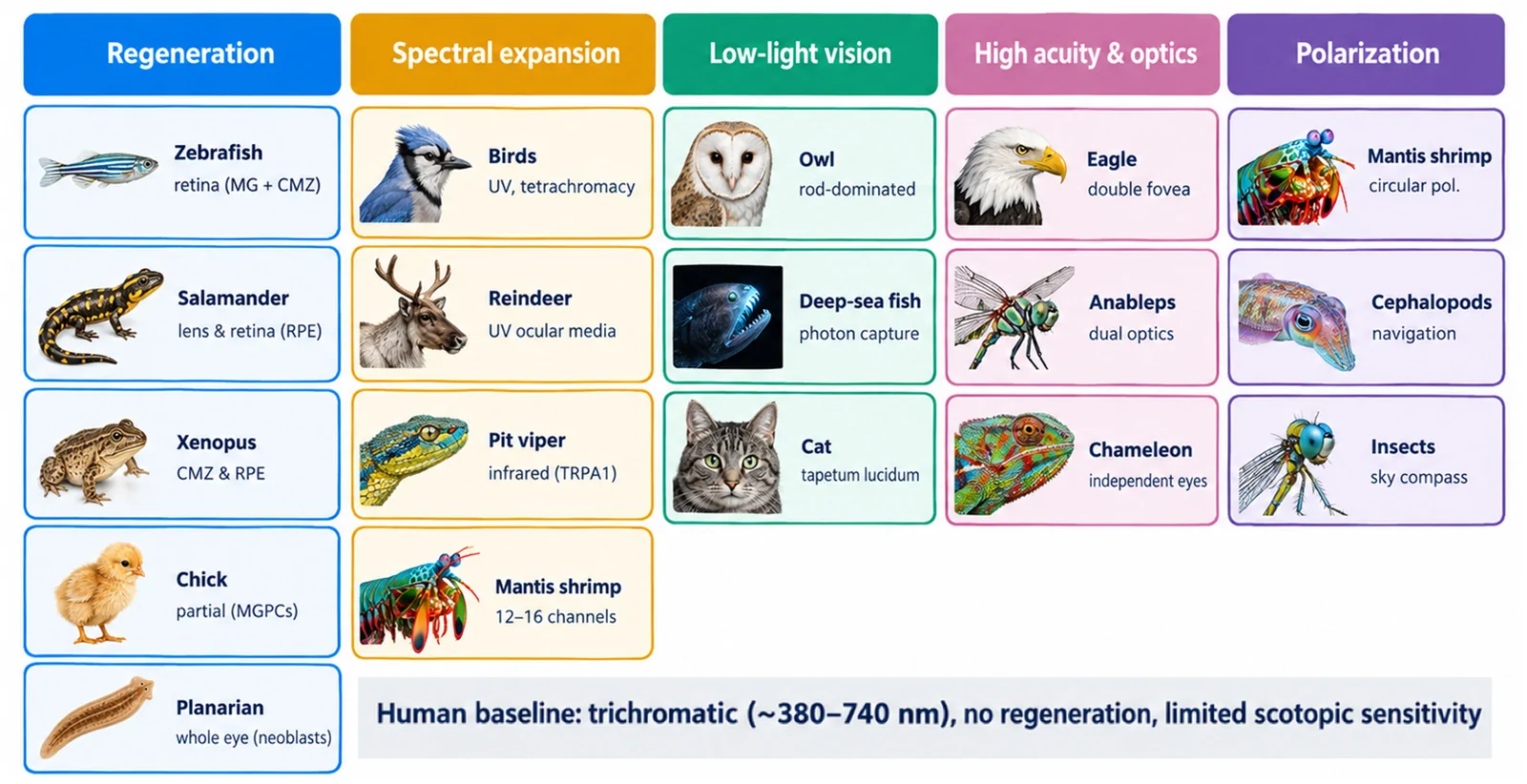
Visual specializations across the animal kingdom. Phylogenetic schematic of representative species and their visual specializations, spanning regeneration (planarian, zebrafish, salamander, Xenopus), spectral expansion (birds, reindeer, snake), low-light adaptation (owl, deep-sea fish, cat), high acuity and optics (eagle, four-eyed fish), and polarization/multi-channel vision (mantis shrimp). Humans provide the trichromatic, non-regenerating baseline.

### 9.2 Controversies and debates

Several debates shape the field. First, the feasibility of mammalian Müller glia reprogramming is contested: while *Ascl1*-based and combinatorial approaches generate new neurons in adult mice, efficiency is low, the regenerated cells are often immature or incompletely integrated, and it remains unproven that they can restore vision in a large, complex human retina (Jorstad et al., 2017; Lahne et al., 2020; Pavlou et al., 2025; Jorstad et al., 2020). Our assessment is that the approach is scientifically valid, but requires substantial gains in efficiency, maturation, and synaptic integration before clinical translation. Second, the relative merits of endogenous stem/progenitor reactivation vs. Müller glia reprogramming vs. transplantation are unresolved; the three strategies target different cell sources and may prove complementary, not competitive (Tropepe et al., 2000; Grise et al., 2021; Mandai, 2023). Third, optogenetics vs. prostheses is an active competition: optogenetics offers cellular resolution and a biological interface but currently requires bright light and goggles; prostheses offer a direct electronic interface but limited resolution and longevity. The likely outcome is a matching of modality to disease stage and patient need. Fourth, the therapy–extension boundary raises the question of where restoration ends and extension beyond native function begins. This boundary is ethically and regulatorily significant, especially for spectral extension and acuity enhancement in healthy individuals.

### 9.3 Research gaps

Key gaps remain. In regeneration, we lack efficient, safe methods to reprogram human Müller glia, to reactivate endogenous progenitors, and to integrate new neurons into functional circuits. For RGC replacement, long-distance axon regeneration, target recognition, and retinotopic mapping remain largely unsolved (Soucy et al., 2023). In spectral function, the neural basis of added channels is poorly understood. Human tetrachromacy carriers show that an extra opsin does not guarantee an extra perceptual dimension (Jordan et al., 2010). In low-light and high-acuity translation, we have detailed biological descriptions, but few concrete interventions that improve these capacities in humans. Across all areas, long-term safety, durability, and functional integration are the dominant unmet needs. Finally, most extension technologies are evaluated in animal models or small human studies; rigorous, standardized functional outcomes are needed to compare approaches.

### 9.4 Vision beyond the native range: evidence and limits

A distinct question is whether nature's specializations can be used not merely to restore lost vision but to extend human vision beyond its native range, into the ultraviolet or infrared, toward superhuman acuity, or toward additional spectral channels. Using the definitions in the Introduction, this is the domain of **extension** and **enhancement**, and the evidence here is thinner. The strongest case is spectral extension via upconversion devices, which has reached proof-of-concept in humans (wearable NIR contact lenses Cai et al., 2019; Do, 2025) and preclinical demonstration in animals (injectable nanoantennae Ma et al., 2019). UV perception through UV-transmitting optics is clinically demonstrated, but is properly considered restoration of a latent capacity. Biological extension of acuity, low-light sensitivity, or channel number remains theoretical, constrained by the need to co-engineer optics, photoreceptors, and neural circuitry. We regard vision extension as a legitimate but early-stage research direction, distinct from the restoration-focused core of this review, and one that warrants cautious, transparent development with attention to the ethical considerations below.

### 9.5 Future directions

The most promising directions combine strategies. Regenerative biology plus optogenetics could rebuild a light-sensitive retina where none remains. Opsin engineering plus upconversion nanophotonics could extend human spectral sensitivity both biologically and optically. AI-driven, bio-inspired image processing could match prosthetic stimulation to the retina's neural code. Single-cell and spatial omics of regenerating species will continue to reveal the gene regulatory logic of repair, while advances in AAV delivery, gene editing, and biomaterials will improve the precision and safety of interventions. The field is moving from mimicking individual biological features to integrating them into systems that restore the whole visual function.

### 9.6 Ethical considerations

Interventions that alter the retina or its optics carry risks of inflammation, atrophy, or unintended visual consequences, as illustrated by chorioretinal atrophy and other complications after gene therapy (Lorenz, 2025). Equitable access is a concern, as advanced therapies and devices are expensive and may widen disparities in visual health. For interventions that extend vision beyond the natural range, infrared vision, added spectral channels, superhuman acuity, the therapy–extension boundary becomes ethically and regulatorily significant, and may create pressures for use in military, occupational, or consumer contexts. Germline editing for non-therapeutic purposes is widely considered unacceptable. We argue that the field should prioritize restoration for those with vision loss, pursue any extension of human vision cautiously and transparently, and engage regulators, patients, and the public early in the development of such technologies.

### 9.7 Conclusion

Nature has produced, many times over, strategies that address the problems limiting human vision. Regenerating species show that a functional retina can be rebuilt; spectral specialists show how the visible band is tuned; nocturnal and deep-sea animals show how to see in near-darkness; raptors and optical specialists show how to maximize resolution; and a range of species show how to extract polarization and process visual information efficiently. The translational task is to borrow these strategies with engineering rigor and biological humility recognizing that each adaptation is part of an integrated system of optics, sensors, and neural circuits.

The past decade has demonstrated that this is possible: gene therapy, optogenetics, and prostheses have restored vision to the blind, and bio-inspired devices have extended human perception. The evidence-grading approach used in this review makes clear which of nature's strategies are already clinical realities, which are advancing through preclinical development, and which remain theoretical. The goal is to move toward a future in which vision loss is reversible.
